# KLF4 regulates skeletal muscle development and regeneration by directly targeting *P57* and *Myomixer*

**DOI:** 10.1038/s41419-023-06136-w

**Published:** 2023-09-18

**Authors:** Shufang Cai, Xiaoyu Wang, Rong Xu, Ziyun Liang, Qi Zhu, Meilin Chen, Zhuhu Lin, Chenggan Li, Tianqi Duo, Xian Tong, Enru Li, Zuyong He, Xiaohong Liu, Yaosheng Chen, Delin Mo

**Affiliations:** State Key Laboratory of Biocontrol, School of Life Sciences, Sun Yat-Sen University, Guangzhou, 510006 Guangdong China

**Keywords:** Differentiation, Mitosis

## Abstract

Krüppel-like factor 4 (KLF4) is an evolutionarily conserved zinc finger-containing transcription factor that regulates diverse cellular processes such as cell proliferation, apoptosis, and differentiation. Our previous study showed that KLF4 expression is upregulated in skeletal muscle ontogeny during embryonic development in pigs, suggesting its importance for skeletal muscle development and muscle function. We revealed here that KLF4 plays a critical role in skeletal muscle development and regeneration. Specific knockout of KLF4 in skeletal muscle impaired muscle formation further affecting physical activity and also defected skeletal muscle regeneration. In vitro, KLF4 was highly expressed in proliferating myoblasts and early differentiated cells. KLF4 knockdown promoted myoblast proliferation and inhibited myoblast fusion, while its overexpression showed opposite results. Mechanically, in proliferating myoblasts, KLF4 inhibits myoblast proliferation through regulating cell cycle arrest protein P57 by directly targeting its promoter; while in differentiated myoblasts, KLF4 promotes myoblast fusion by transcriptionally activating Myomixer. Our study provides mechanistic information for skeletal muscle development, reduced muscle strength and impaired regeneration after injury and unveiling the mechanism of KLF4 in myogenic regulation.

## Introduction

Skeletal muscle, as a highly plastic and dynamic tissue, constitutes approximately 35% of the body weight and plays crucial roles in the maintaining posture, movement, and homeostasis of organisms [1]. Skeletal muscle formation, termed myogenesis, is a multi-step process, including the determination of myogenic progenitors committed into myoblasts, myoblast proliferation, exiting from the cell cycle, and subsequent myoblast differentiation and fusion into multinucleated myotubes [2]. This process is mainly orchestrated by myogenic regulatory factors (MRFs: Myf5, MyoD, MRF4, and myogenin) and MEF2 family transcription factors (MEFA-D) [3]. Their stage-specific expressions and associations with various regulators advance the myogenesis program by activating the transcription of structural and regulatory muscle-specific genes, which is a prerequisite for healthy development of skeletal muscle [4].

Skeletal muscle formation depends on a pool of skeletal muscle progenitor cells (or myoblasts), which continue to proliferate and at same time provide differentiated cells, building the embryonic and fetal muscle masses [4]. In proliferating myoblasts, Myf5 promotes Cyclin D1 expression; MyoD initiates the expression of CDC6 and MCM2, which are primarily involved in making chromatin operational for DNA replication and progression of cells through S-phase. In response to differentiation signals, MyoD and MyoG synergize to upregulate the expression of key regulators of cell cycle exit, including P21 and P57, and repress the activity of Cyclins and Cyclin-dependent kinases [5]. Both the decrease of cell pool expansion caused by the inhibition of myoblast proliferation and the failure of myoblast to exit the cell cycle will lead to abnormal skeletal muscle development [6].

The unique feature of myotubes, the multinucleated syncytium structure, indicates the key significance of myoblast fusion in the process of myogenesis. Many signaling molecules have been implicated in mammalian myoblast fusion, including Rho family small GTPases (RhoA, Rac1, and Cdc42), N- and M-cadherins, focal adhesion kinase (FAK), mitogen-activated protein kinase (MAPKs), Wnt, Myomaker, and Myomixer [7]. Myomaker is required for hemifusion, whereas the subsequent transition from hemifusion to complete fusion depends on Myomixer [8, 9]. Disruption of the genes encoding Myomaker and Myomixer causes embryonic death due to the absence of multinucleated muscle fibers [10, 11].

Krüppel-like factor 4 (KLF4) is a conserved zinc-finger transcription factor of the KLFs family that is characterized by a C-terminal three-zinc-finger DNA-binding domain [12]. KLF4 usually highly expresses in post-mitotic and differentiating epithelial cells in the skin, lungs, and gastrointestinal tracts, and acts as an essential regulator of cell activities such as proliferation, differentiation, and apoptosis [13, 14]. *KLF4*^-/-^ mice die shortly after birth because of defects in the skin barrier function [14]. In vascular smooth muscle, KLF4 was identified as a transcriptional target of bone morphogenetic proteins (BMP-2, −4, and −6) and transforming growth factor-β1 (TGF-β1) to modulate cell differentiation [15, 16]. KLF4 is also one of four factors that reset the fate of somatic cells, reprogramming them as induced pluripotent stem cells [17]. Our previous study showed that KLF4 expression is upregulated in skeletal muscle ontogeny during embryonic development in pigs, suggesting its importance for skeletal muscle development and muscle function [18]. However, it has not been elucidated how KLF4 functions during skeletal muscle development and regeneration.

In the present study, we were surprised to find that KLF4 expression was significantly positively correlated with the expression of myogenic regulators Myog, MyoD, Myf5, and Myf6 in healthy human skeletal muscle samples of various ages, and KLF4 expression was upregulated in the skeletal muscle of Duchenne muscular dystrophy (DMD) mice. These results aroused our interest in KLF4 function in skeletal muscle development and regeneration, and prompted us to carry out in-depth research on this. It was revealed that the conditional ablation of KLF4 in skeletal muscle caused impairment of embryonic and postnatal muscle formation. The loss of KLF4 in satellite cells (SCs) led to blocked myotube formation and defective muscle regeneration. Utilizing KLF4 gain- and loss-of-function studies with C2C12 cells, we demonstrated that KLF4 regulates myoblasts proliferation and fusion by regulating P57 and Myomixer expression.

## Materials and methods

### Mice

*KLF4*^*fl/fl*^ mice carrying floxed KLF4 alleles with loxP sites on both sides of the functional region (contains Extron 3 and Extron 4) of *KLF4* gene were purchased from Cyagen Biosciences Co., Ltd. (Suzhou, China). *Myf5*^*Cre/+*^ (stock #007893) mice were purchased from the Jackson Lab. *KLF4*^*fl/fl*^ mice were crossed with *Myf5*^*Cre/+*^ mice to generate *KLF4*^*fl/fl*^; *Myf5*^*Cre/+*^ mice. Mice were allocated randomly to experimental groups and processed independent on size, body weight or age. All mice used in this study had a C57BL/6 J genetic background and were housed in SPF conditions during the experiment. All experimental procedures involving mice in this study were approved by the Animal Care and Use Committee of Guangdong Province and carried out in accordance with ethical standards.

### Grip-strength test

For measurement of muscle force 8–10-week aged control and KLF4 cKO mice were allowed to grasp onto the horizontal metal grid of the grip strength meter (Columbus Instruments, Columbus, Ohio) by using all fours and pulled backwards 3 times. The force applied to the grid each time before the animal lost its grips was recorded in Newton.

### Exhaustive swimming exercise performance test

8–10-week aged control and KLF4 cKO mice were encouraged to swim for 60 min to adapt to the swimming environment. One week later, the mice were made to swim with a load attached to the tail base equal to 15% of their body weight, in a swimming pool (height: 300 mm, diameter: 260 mm) with warm water (maintained at 25 ± 2 °C). Exhaustive swimming time was recorded as the time when each mouse was unable to return to the surface to breathe within 7 s. The time taken until this point was defined as the exercise endurance value.

### Cardiotoxin (CTX) injury

CTX (Sigma, Shanghai, China) was dissolved in sterile saline to a final concentration of 20 µM. To induce injury, 8–10-week-old female mice were anesthetized using a ketaminexylazine cocktail and the hindlimbs were cleaned with 75% alcohol. Then, using hypodermic syringes, 50 µl of 20 μM CTX was injected into the left and right tibialis anterior (TA) muscles, respectively. Regenerating TA muscles were isolated 3, 10, and 21 days after CTX injection.

### Immunohistochemistry

Freshly isolated regenerating TA muscles and limbs of the control and KLF4 cKO embryos were fixed in 4% paraformaldehyde at 4 °C for 24 h, dehydrated by graded ethanol, and embedded in paraffin. Paraffin-embedded samples were cut into 5 μm sections using rotary microtome (Microm HM 340, Germany). Paraffin sections were rehydrated in graded ethanol, and analyzed by immunostaining with specific antibodies (listed in Table S1) using Mouse on the Mouse Polymer IHC Kit (Abcam, ab269452) as per the manufacturer’s instruction. Images were captured by laser scanning confocal microscope (Leica TCS-SP5, Germany). The myofiber diameters were quantified via Image-Pro Plus6 software.

### Satellite cell isolation and cell culture

Satellite cells (SCs) were isolated by fluorescence-activated cell sorting (FACS) [6]. Hindlimb muscles of 8-week-old mice were minced and digested with 0.3% type II collagenase (Sigma) for 1.5 h. The cell suspension was filtered through a 70 μm cell strainer (biosharp), and then centrifuged at 600 *g* for 5 min. The mononuclear cells were suspended in PBS with 3% FBS for fluorescence staining. Cells were sorted and analyzed by flow cytometry. Markers for SCs isolation were CD31^−^, CD45^−^, CD11b^−^, Sca1^−^, CD34^+^, and Integrin α7 ^+^. Purified SCs were cultured in 24-well plates in Dulbecco’s Modification of Eagle’s Medium (DMEM, Corning) with 20% FBS (Gibco), basic FGF (10 ng/ml, Sigma) and 1% penicillin/streptomycin. Furthermore, when reaching confluence, proliferating cells were incubated with DMEM supplemented with 2% horse serum (Gibco) (differentiation medium, DM) to induce differentiation.

C2C12 cells were purchased from the American Type Culture Collection (ATCC), cultured in DMEM with 10% (v/v) FBS (growth medium, GM). Cells were evenly planked in 6-well plates or 24-well plates with the same cell density, three of which were randomly allocated to control or experimental groups. To induce differentiation, cells were switched into DM after reaching 100% confluence. All cells were cultured in a 37 °C incubator with 5% CO_2_.

### RNA interference and overexpression

For RNA interference, negative control siRNAs (siNC) and four stealth mouse KLF4 siRNAs were purchased from GenePharma Co., Ltd. (Shanghai, China). The sequences of four Klf4-targeting si-RNAs are listed in Table S2, all of which are efficient (Fig. S1). siKLF4, a mixture of siKLF4-1, siKLF4-2, and siKLF4-3, was used in all of the following analysis. For KLF4 expression vector (pcDNA3.1-KLF4), the coding sequences (CDSs) of mouse *KLF4* gene were inserted into pcDNA3.1 vector (Invitrogen). C2C12 cells were seeded into 6- or 12-well plates at 12 h before treatment, and then transfected with siRNAs or plasmids using Lipofectamine 3000 (Invitrogen).

### RNA extraction and Real-time quantitative PCR

Total RNA was extracted from cultured C2C12 cells and regenerating TA muscles using Trizol Reagent (Invitrogen). Then, cDNA was synthesized from 1 μg total RNA using StarScript II First-strand cDNA Synthesis Mix (Genestar, Beijing, China). Real-time quantitative PCR (qPCR) analyses were performed on LightCycler 480 II (Roche, Basel, Switzerland) using SYBR Green qPCR Mix (GDSBio, Guangzhou, China), with GAPDH as an internal control for normalization. Primers are listed in Table S3.

### Western blot

Protein extracts of cultured C2C12 cells or muscle tissues were obtained using RIPA lysis buffer (Solarbio, China) supplemented with protease inhibitor phenylmethanesulfonyl fluoride (PMSF, Thermo Scientific). Total protein was separated by SDS-PAGE and transferred onto PVDF member (Bio-Rad, USA). Then, immunoblotting for target proteins were carried out by specific antibodies (listed in Table S1). Blots were visualized by the chemiluminescence imaging system (BLT, GelView 6000Pro, Guangzhou, China). The grayscale statistics were quantified via Image-Pro Plus6 software.

### Immunofluorescence

Cells cultured onto 24-well plates were fixed in 4% paraformaldehyde, permeabilized in 0.5% Triton X-100, and blocked with 4% BSA/PBS. Then, the cells were incubated with primary antibodies overnight at 4 °C and incubated with secondary antibodies for 1 h at room temperature. Finally, the nuclei were counterstained with DAPI (1:1000 in PBS). Antibodies are listed in Table S1. Immunostaining images were obtained via fluorescent reverse microscopy (Nikon, Japan).

### 5-Ethynyl-2′-deoxyuridine (EdU) assay

EdU assay was performed with an EdU kit (RiboBio, China). C2C12 cells transfected with siRNAs or pcDNA3.1 vectors were seeded onto 24-well plates and cultured in GM for 48 h, then switched into the fresh DMEM medium supplement with EdU (50 mM) and incubated for 2 h. Followed by fixation, permeabilisation, and EdU staining with Apollo567 (RiboBio, China). The cell nuclei were stained with DAPI (1:1000 in PBS).

For newly isolated SCs, cells were seeded onto 24-well plates and cultured in GM for 68 h, then switched into the fresh DMEM medium supplement with EdU (50 mM) and incubated for 4 h. Afterwards, the cells were incubated with Pax7 antibody overnight at 4 °C and incubated with secondary antibodies for 1 h, then stained with Apollo567 for 30 min at room temperature. Finally, the nuclei were counterstained with DAPI.

### Chromatin immunoprecipitation (ChIP)

C2C12 cells transfected with pcDNA3.1 vector or pcDNA3.1-KLF4 were cross-linked with 1% formaldehyde for 8 min generate cross-link of protein-DNA complexes. Cell lysates were sonicated by Bioruptor (Covaris, USA) for 8 min to generate chromatin fragments of 200–300 bp DNA. The clarified nuclear extracts were incubated with KLF4 antibody, tri-methyl-histone H3 (Lys27) antibody, and tri-methyl-histone H3 (Lys4) antibody overnight at 4 °C. IgG was used as a negative control. We used JASPAR (http://jaspar.genereg.net) to predict KLF4 binding sites in *P57* and *Myomixer* promoter sequence (−2000bp-300bp relative to the transcription start site (TSS) of *P57* and *Myomixer* genes, respectively). Specific qPCR primer for each predicted motif was designed on the NCBI database, and the specificity was verified using Mouse genomic DNA. Finally, precipitated chromatin DNA was analyzed via qPCR. The primers that successfully amplify DNA are listed in Table S4.

### Vector construction and dual luciferase reporter assay

The promoter sequences of the mouse *P57* and *Myomixer* genes, including predicted KLF4 binding motifs, were obtained from the NCBI database (www.ncbi.nlm.nih.gov) and amplified by PCR, with the KLF4 binding motif identified by ChIP-qPCR being mutated. The wild-type and mutated promoter were respectively inserted into the pGL3.0-basic vector between the KpnI and HindIII restriction sites using the In-Fusion HD Cloning kit (Takara Bio).

When the density reached 70%, C2C12 cells were transfected with 500 ng of the wild-type or mutated promoter construct mixed with 50 ng of Renilla luciferase vector (pRL-TK) using Lipofectamine 3000 (Invitrogen). The medium was changed after 12 h. We used the Dual Luciferase Reporter Assay System (Promega, Madison, WI) to detect chemiluminescence, and promoter activity was determined by dividing the relative fluorescence value for firefly luciferase by that of Renilla luciferase.

### Statistical analysis

All experiments in this study were performed at least in triplicate. Data are presented as mean ± SD, and the statistical significance analysis was performed using an unpaired two-tailed Student’s *t*-test to test differences between groups. Values of *P* < 0.05 were considered as statistical significance.

## Results

### Conditional deletion of KLF4 causes defect in muscle development

**Fig. 1:**
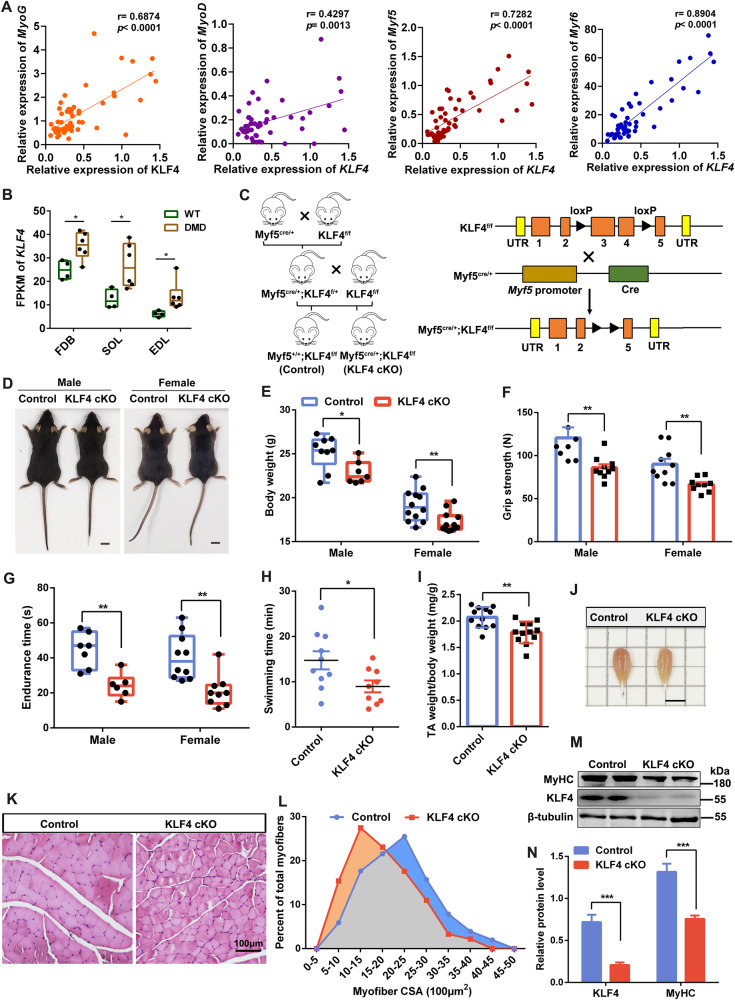
KLF4 is essential for skeletal muscle development and further affects physical activity. **A** Pearson correlation between the expression of KLF4 and several myogenic genes was analyzed using RNA-Seq data (GSE164471) of skeletal muscle samples from 53 healthy individuals of different ages. **B** Comparison of KLF4 expression in flexor digitorum short (FDB), extensor digitorum long (EDL), and soleus (SOL) of Duchenne muscular dystrophy (DMD) mice and wild-type (WT) mice (DMD: *n* = 6; DMD: *n* = 4). N. Data were obtained from the GEO database (GSE162455). **C** Outline of the scheme to obtain control and KLF4 cKO mice. **D** Representative images of 8-week-aged control and KLF4 cKO mice. Scale bar = 1 cm. **E** The body weight of control and KLF4 cKO mice (8-week-aged; control: *n* = 9 (male) and 12 (female); KLF4 cKO: *n* = 7 (male) and 10 (female)). **F**–**H** Skeletal muscle strength and physical activity were evaluated using grip strength (control: *n* = 7 (male) and 10 (female); KLF4 cKO: *n* = 10 (male) and 9 (female)), physical endurance (control: *n* = 7 (male) and 10 (female); KLF4 cKO: *n* = 6 (male) and 9 (female)), and swimming time tests (control: *n* = 10; KLF4 cKO: *n* = 9), respectively. **I** Quantification of TA weight/body weight in control and KLF4 cKO mice (*n* = 6). **J** Representative image of tibialis anterior (TA) muscle from control and KLF4 cKO mice at 8 weeks of age. Scale bar = 0.5 cm. **K** Hematoxylin and eosin (H&E) staining of the TA muscle cross-sections from 8-week-aged control and KLF4 cKO mice. Scale bar = 100 μm. **L** The distribution of fiber size measured by the cross-section area (CSA) in TA muscles from control and KLF4 cKO mice (*n* = 4, each). **M** Western blot detected the protein levels of KLF4 and MyHC in TA muscles from control and KLF4 cKO mice. **N** The relative protein levels of target proteins normalized to β-tubulin signals in (**M**) were obtained through Western blot (WB) band grey scanning. Data are represented as mean ± SD. ^*^P < 0.05, ^**^P < 0.01, ^***^P < 0.001 (Student’s *t* test).

We set out to analyze the Pearson correlation between the expression of KLF4 and myogenic genes using RNA-Seq data of 53 healthy human skeletal muscle samples of various ages (GSE164471) and found the expression of KLF4 was positively correlated with Myog, MyoD, Myf5, and Myf6 (Fig. 1A). Moreover, the expression of KLF4 in skeletal muscles in Duchenne muscular dystrophy (DMD) mice was significantly higher than that in (wild-type) WT mice (GSE162455)(Fig. 1B). To investigate the roles of KLF4 in skeletal muscle development, mice carrying floxed KLF4 alleles with loxP sites (*KLF4*^*fl/fl*^) were crossed with mice expressing Cre recombinase from *Myf5* locus (*Myf5*^*Cre/+*^) to generate *Myf5*^*Cre/+*^*;KLF4*^*fl/fl*^ mouse (hereafter referred to as KLF4 cKO), in which exons 3 and 4 of the *KLF4* gene were knocked out. M*yf5*^*+/+*^*;KLF4*^*fl/fl*^ littermates were used as the control (Fig. 1C). The genotype of mice was identified (Supplementary Fig. S2). KLF4 cKO mice showed a dramatic decrease in the body weight and exhibited significant decreases in the skeletal muscle strength and physical activity (Fig. 1D–H). In addition, the size and weight of TA muscles were lower in KLF4 cKO mice (Fig. 1I, J). We also found that the heterozygous KLF4 knockout (*Myf5*^*Cre/+*^*; KLF4*^*fl/+*^) mice had significantly lower body weights (Fig. S3), TA weights, and grip strengths (Fig. S4). H&E staining showed that the cross-sectional areas (CSA) of myofibers in KLF4 cKO mice were markedly smaller than control mice (Fig. 1K, L). As expected, the expressions of KLF4 and myogenic gene MyHC were significantly reduced in the dorsal muscle of KLF4 cKO mice (Fig. 1M, N). Together, these results indicate that KLF4 is important for skeletal muscle development and further affects physical activity.

**Fig. 2:**
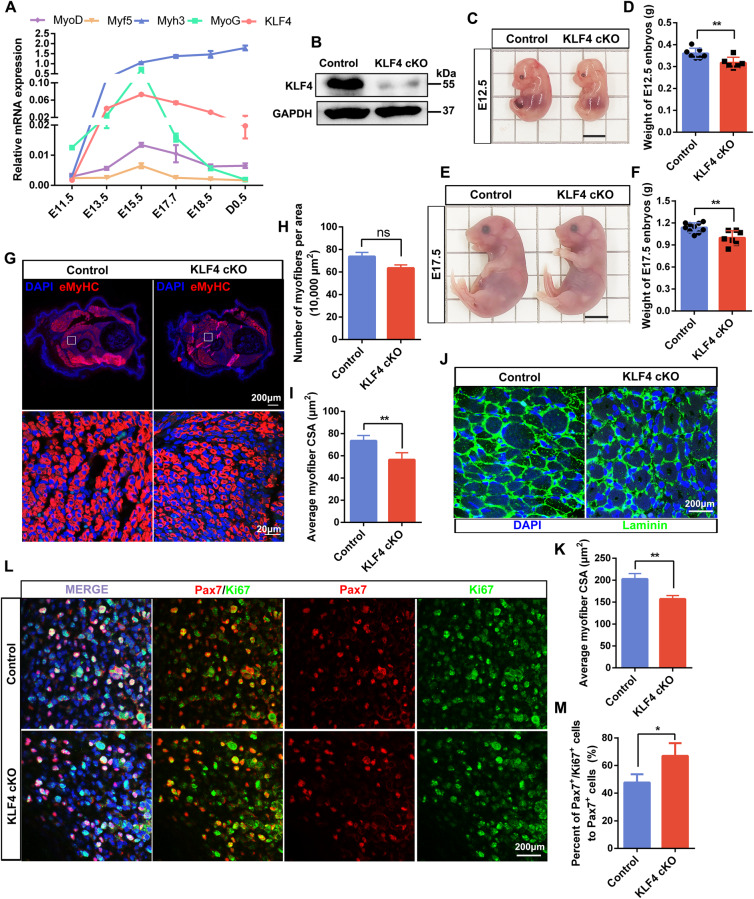
KLF4 is critical for embryonic myogenesis. **A** qPCR measurement of mRNA expression of KLF4 and myogenic markers (Myf5, MyoD, Myogenin, and Myh3) in the dorsal muscle of mouse at several developmental times. GAPDH was used as an internal control for normalization. E: days of embryo age, P: days of age post-birth. **B** WB detected the protein level of KLF4 in limbs of E12.5 embryos. **C** Representative images of control and KLF4 cKO embryos at E12.5. Scale bar = 0.5 cm. **D** Quantifications of the control and KLF4 cKO embryos weight at E12.5 (*n* = 7). **E** Representative images of control and KLF4 cKO embryos at E17.5. Scale bar = 0.5 cm. **F** Quantifications of the control and KLF4 cKO embryos weight at E17.5 (control: *n* = 10; KLF4 cKO: *n* = 7). **G**. Immunofluorescence staining of embryonic myosin heavy chain (eMyHC) was performed on cross sections of limbs of control and KLF4 cKO embryos at E12.5. Nuclei are counterstained with DAPI. Scale bar = 200 μm (top) or 20 μm (bottom). **H**, **I**. Quantifications of the numbers of eMyHC^+^ fibers per area and average myofiber CSA in (G)(*n* = 3). **J** Immunofluorescence staining of Laminin was performed on cross sections of limbs of control and KLF4 cKO embryos at E17.5. Scale bar = 200 μm. **K** Quantifications of average myofiber CSA in (**J**) (*n* = 3). **L** Immunofluorescence staining of Pax7 and Ki67 on cross sections of limbs of control and KLF4 cKO embryos at E12.5. Scale bar = 200 μm. **M** The percentage of Pax7/Ki67 double-positive cells compared with Pax7-positive cells in (**L**) was presented (*n* = 3). Data are represented as mean ± SD. ^*^P < 0.05, ^**^P < 0.01, ^***^P < 0.001; ns not significant (Student’s *t* test).

To determine whether KLF4 also regulates embryonic skeletal muscle development, the expression of KLF4 from embryonic period to newborn was detected. As a result, KLF4 was highly expressed with a peak expression at E15.5, which was consistent with MyoD, Myf5, and MyoG, and suggested its involvement in embryonic myogenesis (Fig. 2A). The protein level of KLF4 was reduced in the dorsal muscle of KLF4 cKO embryos (Fig. 2B). Consistent with adulthood, the weights of KLF4 cKO embryos were significantly less compared to the WT embryos at E12.5 and E17.5 (Fig. 2C–F). Immunofluorescence of eMyHC revealed that although there was no significance in the number of eMyHC^+^ myofibers per area, the CSA of eMyHC^+^ myofibers was significantly decreased in KLF4 cKO limbs at E12.5 (Fig. 2G–I). Further, immunofluorescence of laminin also showed that KLF4 deletion resulted in smaller myofibers at E17.5 (Fig. 2J, K). Interestingly, immunofluorescence for Pax7 and Ki67 revealed that KLF4 deletion accelerated myoblast proliferation (Fig. 2L, M). Cumulatively, these results suggest that KLF4 loss promotes myoblast proliferation but represses myofiber formation.

### KLF4 deletion in myogenic cells impairs skeletal muscle regeneration

**Fig. 3:**
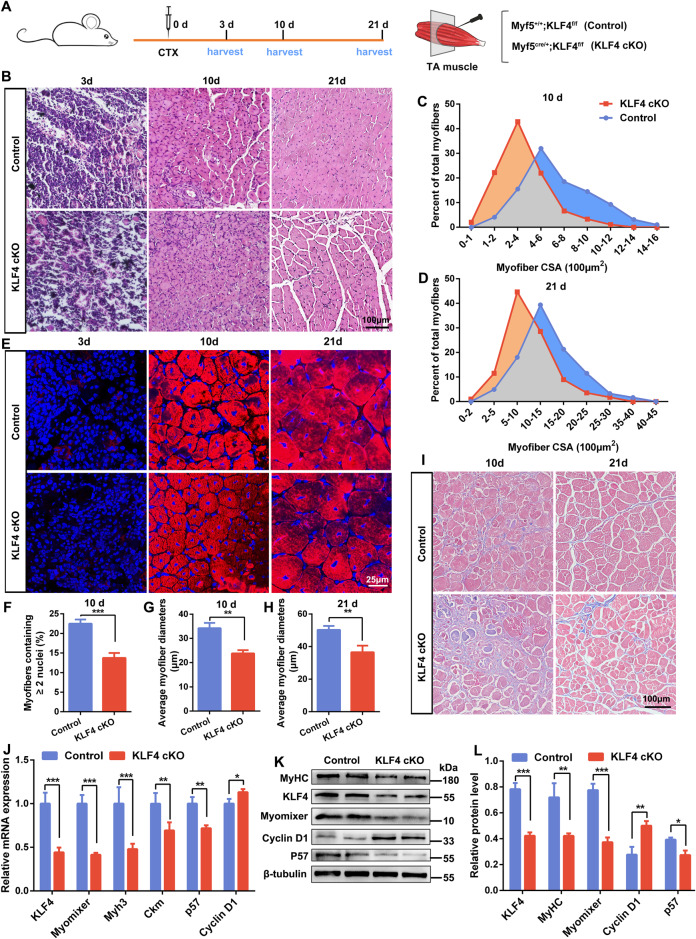
*KLF4* deletion in myogenic cells impairs skeletal muscle regeneration. **A** Experimental design to analyze cardiotoxin (CTX)-induced muscle regeneration in 8–10-week-old control and KLF4 cKO mice. When the TA muscles were treated with CTX, it was defined as day 0. **B** H&E staining on TA muscle sections at 3, 10, and 21 days (3, 10, and 21 d) post-injury (*n* = 3, each). Scale bar = 100 μm. **C**, **D** The distribution of myofiber CSA in regenerating TA muscles at 10 d and 21 d (*n* = 3, each). **E** Immunofluorescence staining of eMyHC was performed on cross sections of TA muscles at 3, 10, and 21 d. Scale bar = 25 μm. **F** The percentage of myofibers containing two or more centrally located nuclei per field at 10 d (*n* = 3). Quantifications of average myofiber diameters at 10 d (**G**) and 21 d (**H**), respectively (*n* = 3, each). **I** Masson trichrome staining was performed on cross sections of TA muscles at 10 d and 21 d. Scale bar = 100 μm. **J** qPCR detection of KLF4, myogenic genes, and cell cycle related genes expression in the TA muscles at 10 d (*n* = 3). **K** WB detected the protein levels of KLF4 and myogenic genes in TA muscles at 10 d. **L** The relative protein levels of target proteins normalized to β-tubulin signals in (**K**) were obtained through WB band grey scanning. Data are represented as mean ± SD. ^*^P < 0.05, ^**^P < 0.01, ^***^P < 0.001 (Student’s *t* test).

Two-month-old control and KLF4 cKO mice were injected with CTX into TA muscles to induce muscle injury and determine whether KLF4 deletion impairs muscle regeneration (Fig. 3A). H&E staining on the cross-sections of TA muscles at 3, 10, and 21 days post-injury revealed a more severe regeneration defect in KLF4 cKO mice. KLF4 cKO muscles exhibited smaller regenerating fibers at 10 and 21 days post-injury (Fig. 3B–D). In line with this, immunofluorescence assay of eMyHC revealed that the number of myofibers containing two or more centrally located nuclei was significantly reduced and the average myofiber diameters decreased markedly in KLF4 cKO mice (Fig. 3E–H). Then, Masson trichrome indicated that collagen-rich fibrous tissue was abundant in the regenerating muscle of KLF4 cKO mice, whereas fibrous tissue was less prolific in the control group (Fig. 3I). Furthermore, the expression levels of Myh3, Ckm, and Myomixer were significantly lower in regenerating muscles from KLF4 cKO mice than control at 10 days post-injury (Fig. 3J–L). These results demonstrate that the loss of KLF4 impairs skeletal muscle regeneration.

### KLF4 regulates the proliferation and differentiation of satellite cells

**Fig. 4:**
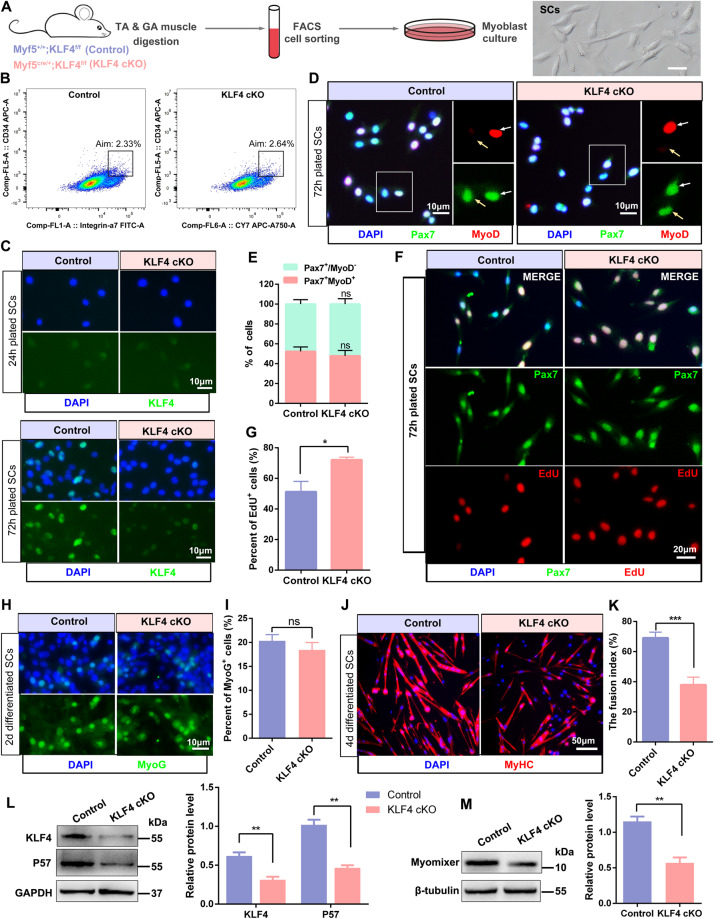
KLF4 regulates the proliferation and differentiation of satellite cells (SCs). **A** Schematic diagram of satellite cells isolation and culture. **B** SCs were isolated by fluorescence-activated cell sorting (FACS) from hindlimb muscles of 8-week-old control and KLF4 cKO mice, and the numbers of SCs were analyzed by flow cytometry (*n* = 3). **C** Primary satellite cells (SCs) were immunostained with KLF4 at 24 h and 72 h after culture. Scale bar = 10 μm. **D** Immunofluorescence staining of Pax7 and MyoD was performed on 24 h-cultured SCs. Scale bar = 10 μm. **E** Quantification of Pax7^+^/MyoD^−^ and Pax7^+^/MyoD^+^ cells in (**D**) (*n* = 3). **F** Representative immunofluorescence of Pax7 in 72 h-cultured SCs having incorporated 5-Ethynyl-2′-deoxyuridine (EdU). Scale bar = 20 μm. **G** Proportions of EdU^+^ SCs 72 h after isolation from control and KLF4 cKO mice (*n* = 3). **H** Immunofluorescence staining of MyoG was performed in 2d-differentiated SCs. Scale bar = 10 μm. **I**. Proportions of MyoG^+^ cells 2d after differentiation (*n* = 3). **J** Immunofluorescence staining of MyHC was performed in 4d-differentiated SCs. Scale bar = 50 μm. **K** The fusion index (the percentage of nuclei in fused myotubes out of the total nuclei) in (**J**) was calculated (*n* = 3). **L**, **M** WB analyses of KLF4, P57 and Myomixer protein expression were performed on 72 h-cultured (for KLF4 and P57) and 4d-differentiated (for Myomixer) SCs isolated from control and KLF4 cKO mice. The relative protein levels of target proteins normalized to GAPDH or β-tubulin signals were obtained through WB band grey scanning.

Satellite cells (SCs) are critical for muscle regeneration after impairment. Therefore, we investigated the importance of KLF4 to SCs. There was no significant change in the number of quiescent SCs between control and KLF4 cKO uninjured TA muscles (Supplementary Fig S5). Consistently, no obvious change was found in the percentage of SCs purified by FACS between control and KLF4 cKO mice, indicating KLF4 might not be necessary for SC maintenance (Fig. 4A, B). Immunofluorescence staining analysis revealed that quiescent and newly activated SCs (24 h in culture) did not express KLF4, while the proliferating WT SCs (72 h in culture) expressed KLF4 with a high level, but cKO SCs’ expression is almost unchanged (Fig. 4C). In addition, there was no difference in the proportions of Pax7^+^/MyoD^-^ cells (quiescent SCs) and Pax7^+^/MyoD^+^ cells (activated SCs), which indicated that KLF4 was not involved in the activation of SCs (Fig. 4D, E). However, SCs from KLF4 cKO mice exhibited increased EdU incorporation, suggesting KLF4 loss promoted SCs proliferation (Fig. 4F, G). Then, we characterized the impact of KLF4 on SCs differentiation and found the proportion of MyoG^+^ cells didn’t change upon KLF4 deletion at 2 d after differentiation (Fig. 4H, I). However, the formation of MyHC^+^ myotubes was impaired, accompanied by a decreased fusion index in KLF4 deletion SCs at 4 d after differentiation (Fig. 4J, K). In line with these results, the expression levels of the cell cycle inhibitor P57 and cell fusion protein Myomixer in KLF4 deletion SCs were decreased during proliferation and differentiation stages, respectively (Fig. 4L, M). Collectively, these results showed that KLF4 loss promoted SCs proliferation but suppressed the fusion of differentiated SCs.

### KLF4 inhibits myoblast proliferation

**Fig. 5:**
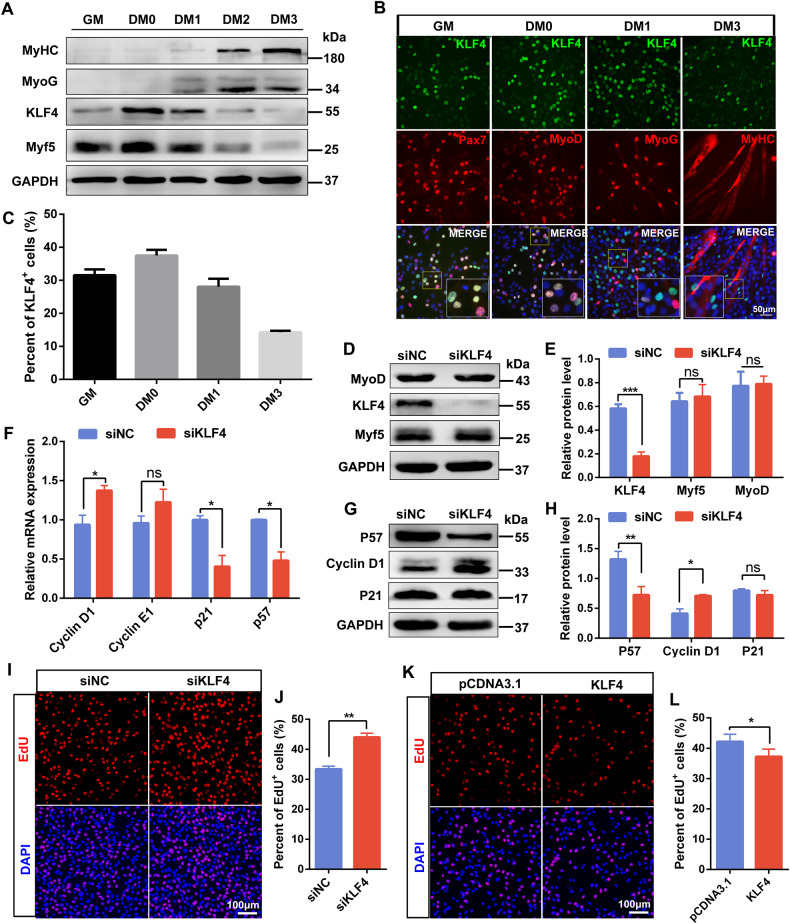
KLF4 inhibits myoblast proliferation. **A** WB analyses of KLF4 and myogenic markers protein expression were performed in C2C12 cells grown in growth medium (GM), as well as in differentiation medium (DM) for 0d, 1d, 2d, and 3d (DM 0-3). GAPDH was used as a loading control. **B** Immunofluorescence staining of KLF4 and myogenic markers in C2C12 cells at several indicated time points. Scale bar = 50 μm. **C** The percentages of KLF4^+^ cells compared with the total number of nuclei in (**B**) were presented (*n* = 3, each). **D** Western blot analyzed the protein levels of KLF4 and two myogenic transcription factors (MyoD and Myf5) in C2C12 cells transfected with negative control siRNAs (siNC) or KLF4 siRNAs (siKLF4). **E** The relative protein levels of target proteins normalized to GAPDH signals in (**D**) were obtained through WB band grey scanning (*n* = 3). **F** qPCR detection of KLF4 and cell cycle-related gene expression in C2C12 cells described as (**D**) (*n* = 3). **G**. WB analyzed the protein levels of KLF4 and cell cycle-related gene expression. **H** The relative protein levels of target proteins normalized to GAPDH signals in (**G**) were obtained through WB band grey scanning (*n* = 3). **I** Representative images of the EdU staining for si-KLF4 C2C12 cells. Scale bar = 100 μm. **J** The percentages of EdU-positive cells compared with the total number of nuclei were presented (*n* = 3). **K**. EdU staining for C2C12 cells after KLF4 overexpression. Scale bar = 100 μm. **L** The percentages of EdU-positive cells in (**K**) were counted (*n* = 3). Data are represented as mean ± SD. ^*^P < 0.05, ^**^P < 0.01, ^***^P < 0.001 (Student’s *t* test).

To study the effects of KLF4 on myogenesis in vitro, the expression profiles of KLF4 were examined during myoblast proliferation and differentiation. WB analysis and immunofluorescence staining demonstrated that KLF4 was highly expressed in proliferating and early differentiated myoblasts, and showed co-expression with Pax7 and MyoD (Fig. 5A–C). Then siKLF4 was employed to knocked down endogenous KLF4 in C2C12 cells and determine the role of KLF4 during myoblast proliferation. The expression of Myf5 and MyoD didn’t show significant difference between KLF4 knockdown and control groups, suggesting that KLF4 does not regulate the fate commitment of myoblasts (Fig. 5D, E), which is in consistent with the above result that KLF4 was not involved in the activation of SCs. In addition, P57 expression was significantly reduced, whereas Cyclin D1 expression was increased in siKLF4 groups (Fig. 5F–H). In line with this, EdU assay revealed the percentages of EdU‐positive proliferating cells increased after knockdown of KLF4 (Fig. 5I, J). On the contrary, KLF4 overexpression decreased the percentages of EdU+ cells (Fig. 5K, L). These results suggested that KLF4 could inhibit myoblast proliferation by increasing P57 expression and repressing Cyclin D1 expression.

### KLF4 inhibits myoblast proliferation by targeting cell cycle arrest protein P57

**Fig. 6:**
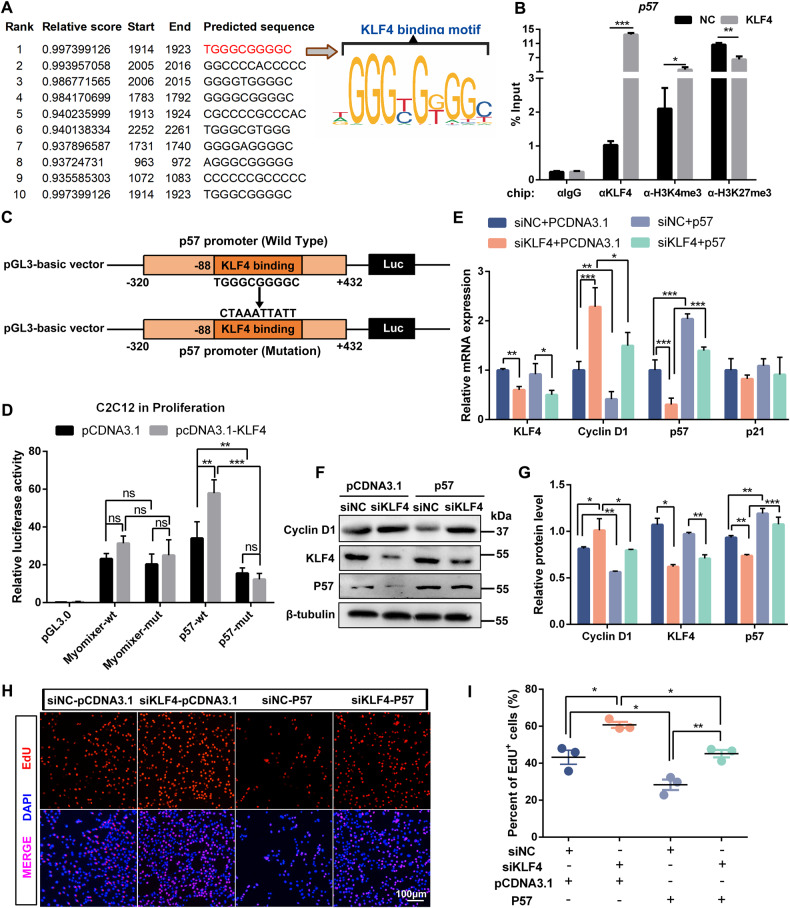
KLF4 targetedly regulates cell cycle arrest protein P57. **A** JASPAR database was used to predict KLF4 binding sites on *P57* promoter (−2000bp ~ +300 bp, relative to the TSS of *P57* gene). The verified sequence containing KLF4-binding motif in this study was emphasized with red. The KLF4 binding sequence logo created in JASPAR was shown. **B** ChIP-qPCR analyses of KLF4, H3K4me3, and H3K27me3 enrichment on *P57* promoter in control and KLF4 overexpression cells. Data were normalized as a percentage of the input. **C** Experimental design to amplify the promoter sequence of *P57* from −320 bp to +432 bp (relative to the TSS of *P57* gene) with the predicted KLF4 binding site (−87bp to −78bp) was mutated, then inserted into the promoter-driven luciferase (Luc) reporter plasmid pGL3 for the dual luciferase assay. **D** The dual-luciferase reporter assays were performed in C2C12 cells transfected with pCDNA3.1 vector or pCDNA3.1-KLF4 vector using reporter plasmids containing the wild type or mutated *P57* promoter (*n* = 3). **E** C2C12 cells, cotransfected with si-NC or si-KLF4 and pcDNA3.1 or pcDNA3.1-P57 vector, were cultured in GM for 48 h. The mRNA levels of KLF4, P21, P57, and Cyclin D1 in four groups were detected by qPCR (*n* = 3). **F** The protein levels of KLF4, P57, and Cyclin D1 were detected by WB in cells described as (**E**). **G** The relative protein levels of target proteins normalized to β-tubulin signals in (**F**) were obtained through WB band grey scanning. **H** C2C12 cells were treated as indicated in (**E**), and EdU staining was performed to compare cell proliferation ability between four experimental groups. Scale bar = 100 μm. **I** The percentage of EdU-positive cells in (**H**) was counted in six microscopic fields for each group (*n* = 3). Data are represented as mean ± SD. *P < 0.05; **P < 0.01; ^***^P < 0.001 (Student’s *t* test).

Considering that above results have demonstrated that KLF4 inhibited cell proliferation by activating the expression of cell cycle inhibitors, such as P21 and P27, we speculated that KLF4 might repress myoblast proliferation by directly regulating P57 expression. To address this notion, we analyzed the P57 promoter using JASPAR database, and found potential KLF4 binding sites upstream the TSS (Fig. 6A). Subsequently, the binding site of KLF4 on −87bp ~ −78bp (relative to the TSS of P57 gene) was verified using ChIP-qPCR. It showed that KLF4 overexpression increased the enrichment of trimethylation of histone 3 lysine 4 (H3K4me3) and decreased the enrichment of trimethylation of histone 3 lysine 27 (H3K27me3) on KLF4 binding site (Fig. 6B). The H3K27me3 marks transcriptionally silenced genes, whereas H3K4me3 is generally associated with active gene expression. Hence, these results revealed that KLF4 promotes P57 transcription by targeting its promoter. In order to further demonstrate the significance of this binding site for transcriptional activity of P57, the promoter sequence from −320bp to +432 bp harboring the potential binding site was amplified, then inserted into reporter constructs for dual luciferase assay (Fig. 6C). The results revealed that the transcriptional activity of P57 was significantly decreased, and the overexpression of KLF4 had no promoting effect on P57 transcription when the KLF4 binding site was mutated (Fig. 6D). To further test whether the effect of KLF4 on myoblast proliferation was mediated by P57, pcDNA3.1-EGFP or pcDNA3.1-P57 vectors were transfected into C2C12 cells simultaneously treated with si-NC or si-KLF4. As predicted, P57 overexpression weakened the promoted expression of Cyclin D1 (Fig. 6E–G) and the accelerated proliferation caused by KLF4 knockdown in C2C12 cells (Fig. 6H, I). These results further proved that KLF4 restrains the proliferation capacity of C2C12 cells by regulating the expression of p57.

### KLF4 is required for myoblasts fusion

**Fig. 7:**
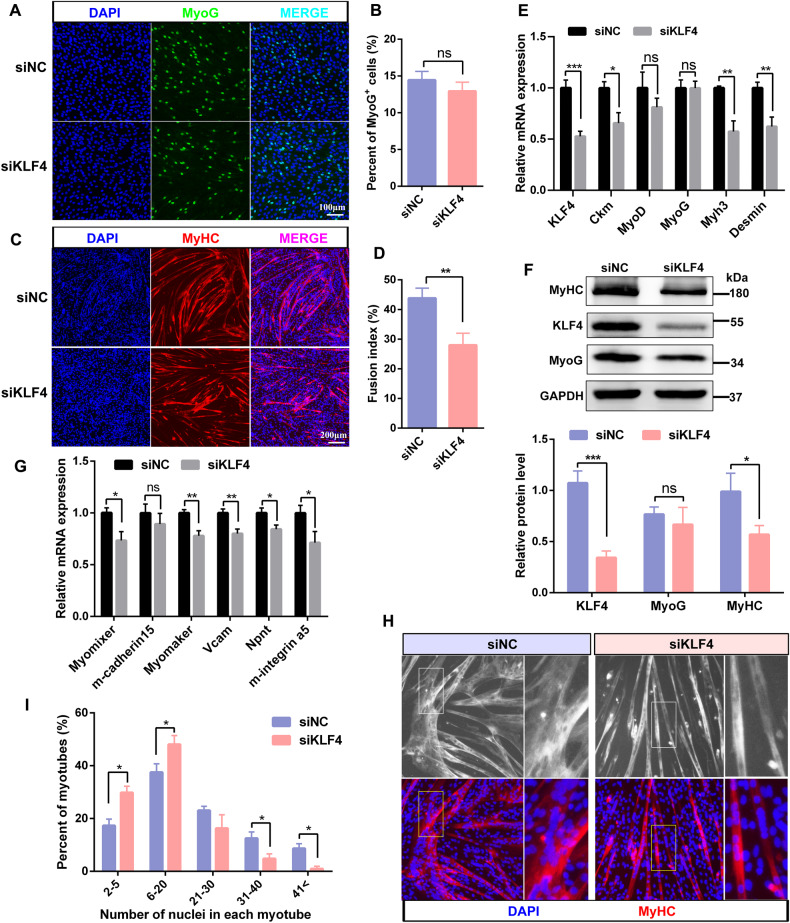
KLF4 is required for myoblast fusion. **A** C2C12 cells were transfected with siNC or siKLF4 for 12 h in GM and then cultured in DM for 1 day followed by immunostaining for MyoG. Scale bar = 100 μm. **B** The percentages of MyoG^+^ cells in (**A**) were counted (*n* = 3). **C** Representative images of immunofluorescence staining for MyHC in C2C12 cells transfected with siKLF4 and differentiated for 3 d. Scale bar = 200 μm. **D** Quantification of the fusion index shown in the (**C**) (*n* = 3). **E** The mRNA levels of KLF4 and myogenic genes in C2C12 cells transfected with siKLF4 and differentiated for 3 d (*n* = 3). **F** The protein levels of KLF4 and myogenic genes in C2C12 cells described in (**E**) (*n* = 3). **G** The mRNA levels of cell fusion-related genes in siKLF4 C2C12 cells differentiated for 3 d (*n* = 3). **H** Representative images of immunofluorescence staining for MyHC in differentiated C2C12 cells. **I** Quantification of the percentage of MyHC^+^ myotubes with the indicated number of nuclei (*n* = 3). Data are represented as mean ± SD. ^*^P < 0.05, ^**^P < 0.01, ^***^P < 0.001 (Student’s *t* test).

C2C12 cells transfected with siNC or siKLF4 were induced to differentiate to evaluate whether KLF4 affected the differentiation and fusion of myoblasts. Immunofluorescence assay showed that knockdown of KLF4 had no effect on the percentage of cells expressing MyoG at DM 1d (Fig. 7A, B) but impaired the formation of MyHC^+^ myotubes, with a reduced fusion index at DM 3d, 5d, and 7d, respectively (Fig. 7C, D and Fig. S6). Consistently, the MyoG expression didn’t changed, while the expression levels of MyHC and other myotube markers including Ckm and Desmin were significantly reduced in siKLF4 group (Fig. 7E, F). This result led us to explore whether KLF4 affects myotube formation by influencing myoblast fusion. To address this question, we detected the expression of gene related to cell fusion and found that the mRNA levels of Myomixer, Myomaker, Vcam, Npnt, and m-integrin α5 were significantly decreased in siKLF4 cells (Fig. 7G). Meanwhile, KLF4 knockdown cells displayed thinner and smaller myotubes with less nuclei than the control cells at DM 3d (Fig. 7H, I). In contrast, overexpressing KLF4 enhanced the expression of MyHC and fusion-related gene in C2C12 cells, accompanied by elevated fusion index (Fig. S7A–E). Collectively, these results strongly suggest that KLF4 is involved in the regulation of myoblast fusion with marginal effects on the expression of MRFs.

### KLF4 directly binds to the myomixer promoter and activates its transcription

**Fig. 8:**
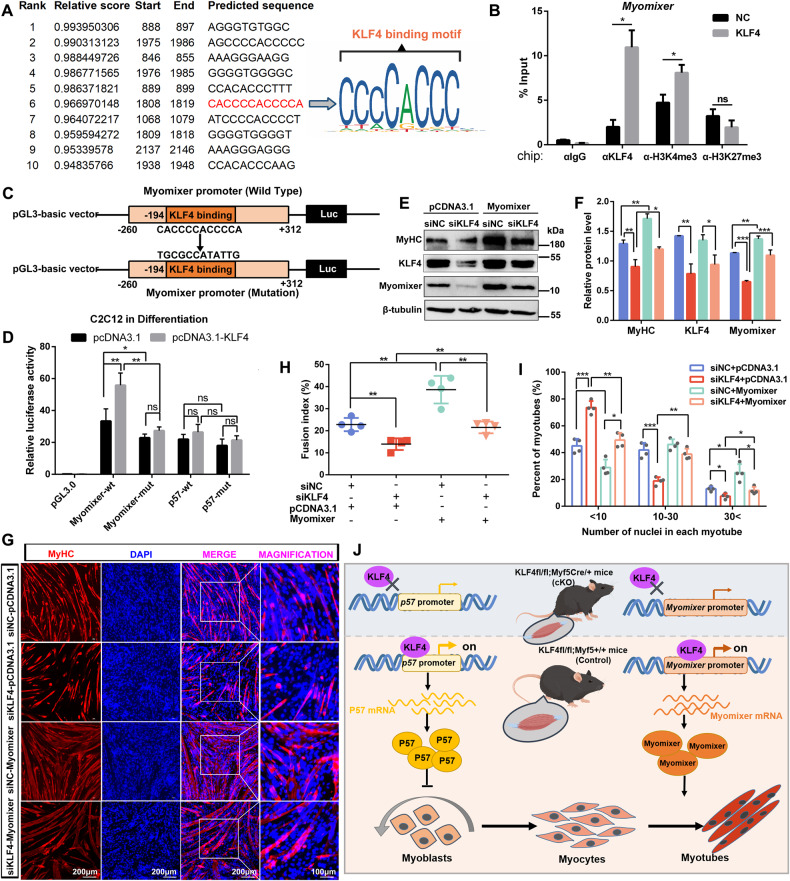
KLF4 directly binds to the *Myomixer* promoter and activates its transcription. **A** JASPAR database was used to predict KLF4 binding sites on *Myomixer* promoter (−2000bp ~ +300 bp, relative to the TSS of *Myomixer* gene). The verified sequence containing KLF4-binding motif in this study was emphasized with red. The sequence logo created in JASPAR was shown. **B** ChIP-qPCR analyses of KLF4, H3K4me3, and H3K27me3 enrichment on *Myomixer* promoter in control and KLF4 overexpression cells. Data were normalized as a percentage of the input. **C** Experimental design to amplify the promoter sequence of *Myomixer* from −260 bp to +312 bp (relative to the TSS of *Myomixer* gene) with the predicted KLF4 binding site was mutated, then inserted into the promoter-driven luciferase (Luc) reporter plasmid pGL3 for the dual luciferase assay. **D** The dual-luciferase reporter assays were performed in C2C12 cells transfected with pCDNA3.1 vector or pCDNA3.1-KLF4 vector and had differentiated for 2 d in DM, using reporter plasmids containing the wild type or mutated *Myomixer* promoter (*n* = 3). **E** C2C12 cells, cotransfected with si-NC or si-KLF4 and pcDNA3.1 or pcDNA3.1-Myomixer vector, were induced to differentiation in DM for 3 d. The protein levels of KLF4, MyHC, and Myomixer were detected by WB. **F** The relative protein levels of target proteins normalized to β-tubulin signals in (**E**) were obtained through WB band grey scanning. **G** C2C12 cells were treated as indicated in (**E**), and MyHC immunofluorescence staining was performed to compare myoblast fusion between four experiment groups. **H**, **I**. The fusion indexes and number of nuclei in each myotube and in (**G**) were quantified in six microscopic fields for each group (*n* = 3). **J** Schematic of KLF4 regulates myoblast proliferation and fusion via targeting the promoters of P57 and Myomixer. Data are represented as mean ± SD. ^*^P < 0.05; ^**^P < 0.01; ^***^P < 0.001 (Student’s *t* test).

Considering that the expression of myomixer which promotes myoblast fusion, was down-regulated in KLF4-knockdown myoblasts, we further investigated whether KLF4 could directly regulate Myomixer transcription. JASPAR was used to predict KLF4 binding sites on myomixer promoter (Fig. 8A). ChIP assays were performed to determine whether Myomixer is the direct transcriptional target of KLF4. It showed that KLF4 bound to the promoter region of Myomixer (harboring the predicted binding site −193bp to −182bp). It was also demonstrated that KLF4 overexpression promoted the enrichment of H3K27me3 on the Myomixer promoter, while inhibited the enrichment of H3K4me3 (Fig. 8B). To confirm the KLF4 binding site is critical for the regulation of Myomixer transcription, the promoter sequence of Myomixer from −521 to +50 harboring the binding motif was amplified, then inserted into reporter constructs for the dual luciferase assay (Fig. 8C). As a result, mutation of KLF4 biding-motif sequence markedly decreased Myomixer promoter activity, indicating KLF4 promotes myoblast fusion by directly activating Myomixer (Fig. 8D). Myomixer overexpression rescued the repressed expression of myotube marker MyHC (Fig. 8E, F) and the impaired myoblast fusion caused by KLF4 knockdown in C2C12 cells (Fig. 8G–I). These results further proved that KLF4 promotes the fusion of C2C12 cells by directly regulating the expression of Myomixer.

## Discussion

Previous studies have demonstrated a correlation between KLF4 expression and a process of growth arrest. For example, constitutive expression of KLF4 in fibroblasts or colon cancer cells resulted in the inhibition of DNA synthesis [19]. This may in part be explained by the ability of KLF4 to activate the *P21* promoter [20], a cell cycle inhibitor. In corneal epithelial cells, KLF4 regulates cycle progression by suppressing canonical TGF-β signaling and upregulating cell cycle inhibitors P16 and P27. Similarly, the present study showed that the expression level of *P57* is significantly decreased in C2C12 cells, accompanied by an increase in the proportion of EDU^+^ cells, due to the knockdown of KLF4. Mechanically, KLF4 inhibits myoblast proliferation by targeting the motif GGGCGGGGC (−87bp to −78bp relative to TSS) on *P57* promoter and activating *P57* transcription. Nevertheless, some studies showed that KLF4 can also facilitate cell proliferation. For example, KLF4 plays an essential role in activation-induced B cell proliferation and B cell development by modulating Cyclin D2 expression [21]. KLF4 also functions as an oncogene to promote the proliferation of bladder cancer and breast cancer cells in the presence of RASV12-Cyclin-D1 signaling or the absence of P21 [22]. Altogether, our findings further support the idea that KLF4 may exert distinct functions to regulate cell proliferation in a tissue context-dependent manner.

In recent years, the functions of KLFs in muscle tissues have become a research hotspot in the field of life science. In cardiac muscle, KLF4 is involved in the negative regulation of cardiac hypertrophy [23]. In vascular smooth muscle, the post-translated modification of KLF4 is regulated by positive factors of cell proliferation and differentiation and plays important roles in the regulation of the vascular smooth muscle phenotype [15, 24]. Here, in skeletal muscle, KLF4 is critical for embryonic myogenesis. Conditional deletion of KLF4 causes defects in muscle development and a significant decrease in skeletal muscle strength and physical activity. KLF4 knockdown didn’t affect the expression of MyoD and MyoG, key regulators of myogenic differentiation, indicating that KLF4 was not involved in the regulation of myoblast differentiation. Myomaker, Myomixer, Vcam, Npnt, and m-integrin α5 are crucial for myogenic cells to fuse [25]. Down-regulated expression of these genes and the thinner and smaller myotubes with fewer nuclei in KLF4 knockdown cells demonstrated that KLF4 promotes myoblast fusion. In this study, Chip-qPCR and dual luciferase reporter assay revealed that KLF4 promoted the transcription of *Myomixer* by targeting its promoter, thus regulating myoblast fusion. Taken together, our study underscored the key functions of KLF4 in regulating skeletal muscle development.

In addition to being involved in embryonic myogenesis, KLF4 is also involved in muscle repair. In the current study, we found KLF4 participated in regulating SC functions. KLF4-deficient SCs failed to form multinucleated myofibers, as evidenced by the reduction of myofibers containing two or more centrally located nuclei in regenerating muscles and the decreased fusion index for FACS-sorted SCs. It is widely known that Myomixer is a key factor in myoblast fusion and myofiber formation [26]. Especially, skeletal muscle does not develop normally in the absence of Myomixer [27]. Here, the repressed expression of Myomixer and eMyHC in TA led to impaired muscle regeneration in KLF4 cKO mice. Muscle regeneration relies on the activation and proliferation of SCs. Previous studies showed that KLF4 was required for embryonic stem cell self-renewal and for maintaining the population of epidermal stem cells [28, 29]. In KLF4 knockdown embryos, the numbers of p63+ stem cells and keratinocyte progenitors were reduced [28]. However, we found loss of KLF4 had no obvious effect on the number of quiescent SCs in uninjured muscle and the proportion of Pax7^+^/MyoD^+^ cells in SCs cultured in vitro, indicating KLF4 was not involved in the maintenance and activation of SCs. Further investigation showed that the EdU incorporation was increased in SCs from KLF4 cKO mice. This is consistent with the finding that KLF4 inhibition promotes the expansion of keratinocyte precursors from adult human skin [30].

In vertebrates, post-translational modifications of histones, such as methylation and acetylation, are extensively used to ensure the temporal and spatial expressions of key genes during tissue-specific development [31]. Lysine methylation on histone 3 (H3) is an important part of the epigenetic regulation network and integrates both cooperative and antagonistic modifications [32]. The ChIP-qPCR assays showed that KLF4 overexpression increased the enrichment of H3K4me3 and decreased the enrichment of H3K27me3 on KLF4 binding sites of *P57* and *Myomixer* promoters. This suggests that KLF4 promotes the transcriptional activation of *P57* and *Myomixer*. Biochemical studies using both cell and animal models revealed that KLFs can mediate sequence-specific regulation of promoters by recruiting distinct histone-modifying enzyme complexes, including p300, CREB-binding protein (CBP), C-terminal binding protein, and histone methyltransferases, to GC-rich regions of promoters [33, 34]. Thus, we speculate that KLF4 couples to histone methyltransferase chromatin remodeling pathways to transcriptionally regulate *P57* and *Myomixer* in myogenic cells.

In conclusion, as illustrated in Fig. 8J, our results implicate that KLF4 is a positive regulator of myogenesis that is essential for skeletal muscle development and regeneration. KLF4 restrains myoblast proliferation by targeting the binding motif GGGCGGGGC on *P57* promoter and activating *P57* transcription. On the other hand, KLF4 promotes the transcription of Myomixer by targeting its binding motif CCCCACCC, thus regulating myoblast fusion. Our findings represent novel discoveries in skeletal muscle formation and provide new insight for future therapy aimed at treating muscle deficiencies or malignant diseases.

## Supplementary information

Supplementary Tables

Supplementary Figure Legends

Supplementary Figure 1

Supplementary Figure 2

Supplementary Figure 3

Supplementary Figure 4

Supplementary Figure 5

Supplementary Figure 6

Supplementary Figure 7

Original Data File

## Data Availability

In this study, we downloaded public RNA-Seq dataset of human skeletal muscle at different ages under accession number GSE164471, and downloaded public RNA-Seq dataset of skeletal muscle from wild-type and Duchenne muscular dystrophy (DMD) mice with an accession number GSE162455.
