# Supplementary material for: KLF4 regulates skeletal muscle development and regeneration by directly targeting P57 and Myomixer
Source: Cell Death Dis. 2023 Sep 18;14(9):612. doi: 10.1038/s41419-023-06136-w (PMC10507053; doi:10.1038/s41419-023-06136-w)
Supplement: Supplementary file 1 — Supplementary Tables [file 41419_2023_6136_MOESM1_ESM.docx]

| Table S1 Antibodies | | |
| --- | --- | --- |
| Antibody name | Catalogue Number | Brand name |
| Anti-rabbit IgG, HRP-linked Antibody | #7074 | Cell Signaling |
| Anti-mouse IgG, HRP-linked Antibody | #7076 | Cell Signaling |
| Anti-mouse IgG (H+L),F(ab')2 Fragment (Alexa Fluor 555 Conjugate) | #4409 | Cell Signaling |
| Anti-rabbit IgG(H+L),F(ab')2 Fragment (Alexa Fluor 488 Conjugate) | #4412 | Cell Signaling |
| Anti-Fast Myosin Skeletal Heavy chain Antibody | Ab91506 | abcam |
| Anti-Ki67 antibody | ab15580 | abcam |
| Anti-Myf5 antibody | ab125301 | abcam |
| Anti-P57 antibody | ab75974 | abcam |
| Anti-P21 antibody | Ab109199 | abcam |
| Anti-Myomixer antibody | AF4580-SP | R&D |
| Anti-Myogenin Antibody | Ab1835 | abcam |
| Anti-GAPDH antibody | ab8245 | abcam |
| Anti-KLF4 antibody | Ab214666 | abcam |
| Anti-KLF4 antibody | AF3158 | R&D |
| Tri-Methyl-Histone H3 (Lys4) (C42D8) Rabbit mAb | 9751T | Cell Signaling |
| Tri-Methyl-Histone H3 (Lys27) (C36B11) Rabbit mAb | #9733 | Cell Signaling |
| Anti-Cyclin D1 antibody | 2978T | Cell Signaling |
| β-Tubulin Antibody | #2148 | Cell Signaling |
| Anti-MyoD antibody | ab212662 | abcam |
| Anti-Pax7 antibody | PA1117 | thermo |
| Anti-Pax7 antibody | PAX7 | DSHB |
| Biotin anti-mouse/human CD11b | 101204 | Biolegend |
| Biotin anti-mouse CD31 | 102404 | Biolegend |
| Biotin anti-mouse CD45 | 103104 | Biolegend |
| Biotin antimouse Ly−6A/E | 108103 | Biolegend |
| StreptavidinAPC/Cyanine7 | 405208 | Biolegend |
| Alexa Fluor 647 antimouse CD34 | 152205 | Biolegend |
| Anti-Integrin α7- FITC | K0046−4 | MBL |

| Table S2 The sequences of KLF4-targeting si-RNAs | |
| --- | --- |
| siRNA | Sequence |
| siKLF4 -1 | F: CCUCCUGGACCUAGACUUUTT |
|  | R: AAAGUCUAGGUCCAGGAGGTT |
| siKLF4 -2 | F: GGUCAUCAGUGUUAGCAAATT |
|  | R: UUUGCUAACACUGAUGACCTT |
| siKLF4 -3 | F: CCACCCACACUUGUGACUATT |
|  | R: UAGUCACAAGUGUGGGUGGTT |
| siKLF4 -4 | F: CACCCACACUUGUGACUAUTT |
|  | R: AUAGUCACAAGUGUGGGUGTT |

| Table S3 The primers for qPCR | | |
| --- | --- | --- |
| Gene | Sequences | |
| Myf5 | F: CCTGTCTGGTCCCGAAAGAAC | R: GACGTGATCCGATCCACAATG |
| MyoD | F: GCCTGAGCAAAGTGAATGAG | R: GCAGACCTTCGATGTAGCG |
| Myh3 | F: AGAAGGAGGAGGCAACTTCTG | R: ACATACTCATTGCCGACCTTG |
| Myogenin | F: GCAATGCACTGGAGTTCG | R: ACGATGGACGTAAGGGAGTG |
| KLF4 | F:TACCCTCCTTTCCTGCCAGA | R: TTTGCCACAGCCTGCATAGT |
| GAPDH | F: CATGGCCTTCCGTGTTCCTA | R: TGCCTGCTTCACCACCTTCT |
| Ckm | F: CTGACCCCTGACCTCTACAAT | R: CATGGCGGTCCTGGATGAT |
| Desmin | F: GTGGATGCAGCCACTCTAGC | R: TTAGCCGCGATGGTCTCATAC |
| Cyclin E1 | F: ATGTCAAGACGCAGCCGTTTA | R: GCTGATTCCTCCAGACAGTACA |
| Cyclin D1 | F: GCGTACCCTGACACCAATCTC | R: CTCCTCTTCGCACTTCTGCTC |
| Myomaker | F: TTCCTCCCGACAGTGAGCAT | R: GCACAGCACAGACAAACCAG |
| P21 | F: CCTGGTGATGTCCGACCTG | R: CCATGAGCGCATCGCAATC |
| P57 | F: CGAGGAGCAGGACGAGAATC | R: GAAGAAGTCGTTCGCATTGGC |
| Vcam | F: AGTTGGGGATTCGGTTGTTCT | R: CCCCTCATTCCTTACCACCC |
| Npnt | F: GAAGCCTCGGCCCTGTAAG | R: AGCATGTATCCGTTGAGACAGTA |
| m-cadherin 15 | F: AGAGAACCCACTGAGGACGA | R: GCCAGTCTTCTGGGTCGTAG |
| m-integrin α5 | F: ATCTGTGTGCCTGACCTG | R: AAGTTCCCTGGGTGTCTG |
| Myomixer | F: GTTAGAACTGGTGAGCAGGAG | R: CCATCGGGAGCAATGGAA |

| Table S4 qPCR primers for ChIP-qPCR | |
| --- | --- |
| Gene | Sequences |
| *Myomixer* (-245 to -146 bp) | F:TTCAGGTCACAGGGCTAGAAAG |
|  | R: AGAAGACAAGAGGGGCCATGT |
| *P57* (-121 to -4 bp) | F: AGCCAATGAGCGTGGCG |
|  | R: GGGCCTCCTCACGATTAGC |
