# Supplementary material for: KLF4 regulates skeletal muscle development and regeneration by directly targeting P57 and Myomixer
Source: Cell Death Dis. 2023 Sep 18;14(9):612. doi: 10.1038/s41419-023-06136-w (PMC10507053; doi:10.1038/s41419-023-06136-w)
Supplement: Supplementary file 2 — Supplementary Figure Legends [file 41419_2023_6136_MOESM2_ESM.docx]

**KLF4 regulates skeletal muscle development and regeneration by directly targeting *P57* and *Myomixer***

**Authors:** Shufang Cai ^1, †^, Xiaoyu Wang ^1, †^, Rong Xu ^1^, Ziyun Liang ^1^, Qi Zhu ^1^, Meilin Chen ^1^, Zhuhu Lin ^1^, Chenggan Li ^1^, Tianqi Duo ^1^, Xian Tong ^1^, Enru Li ^1^, Zuyong He ^1^, Xiaohong Liu ^1^, Yaosheng Chen ^1^, Delin Mo ^1, *^

**Affiliation:** *^1^ State Key Laboratory of Biocontrol, School of Life Sciences, Sun Yat-Sen University, Guangzhou 510006, Guangdong, China.*

^†^ Shufang Cai and Xiaoyu Wang contributed equally to this work and therefore share first authorship.

*** Corresponding author:**

Delin Mo, E-mail: [modelin@mail.sysu.edu.cn](mailto:modelin@mail.sysu.edu.cn); Tel.: +020-39332991.

**Supplementary Figure Legends**

**Figure S1. knockdown efficiency determination of si-RNAs against KLF4.** Four si-RNAs (siKLF4-1, siKLF4-2, siKLF4-3, and siKLF4-4) against MLL1 and control siNC were transfected into C2C12 cells for 2 days respectively, and knockdown efficiency were determined by qPCR. Data are presented as mean ± S.D.; *n* = 3; **p* < 0.05, ***p* < 0.01, ****p* < 0.001 (Student’s *t* test).

**Figure S2. The genotype of mice was identified by PCR.** A. KLF4^fl/fl^ genotype was identified. MT: 200 bp; WT: 137 bp. B. Myf5^cre/+^ genotype was identified. MT: 240 bp; WT: 120 bp.

**Figure S3. Comparison of adult body weight between the control mice and Myf5^cre/+^;KLF4^fl/+^ mice.** A. Representative images of control and Myf5^cre/+^;KLF4^fl/+^ mice in 4 months. Scale bar = 1 cm. B. The body weight of control and KLF4 cKO mice (4 months of age, control: *n* = 6 (male) and 4 (female); Myf5^cre/+^;KLF4^fl/+^: *n* = 6 (male) and 5 (female) ).

**Figure S4. Comparison of grip strength and tibialis anterior (TA) muscle weight between the control mice and Myf5^cre/+^;KLF4^fl/+^ mice.** A. Comparison of grip strength between the control mice and Myf5^cre/+^;KLF4^fl/+^ mice (4 months of age, n ≥ 4). B. Representative image of tibialis anterior (TA) muscle from control and Myf5^cre/+^;KLF4^fl/+^ mice at 4 months of age. C. Quantification of TA weight/body weight in control and Myf5^cre/+^;KLF4^fl/+^ mice (*n* = 6).

**Figure S5. There was no significant change in the number of quiescent satellite cells in** **control and KLF4 cKO uninjured TA muscles.** Immunofluorescence staining of Pax7 was performed on TA muscle cross sections of the control and KLF4 cKO mice at 8 weeks of age. Then, the number of Pax7^+^ cells per area was counted.

**Figure S6. KLF4 is required for myoblast fusion.** A. Representative images of immunofluorescence staining for MyHC in C2C12 cells transfected with siKLF4 and differentiated for 5 d and 7d. Scale bar = 200 μm. B. Quantification of the fusion index shown in the (A).

**Figure S7.** **KLF4 overexpression promoted myoblast fusion.** A. The mRNA levels of KLF4 and myogenic genes in C2C12 cells transfected with pCDNA3.1-KLF4 vector and differentiated for 3 d. B. The protein levels of KLF4 and myogenic genes in C2C12 cells described in (A). C. Representative images of immunofluorescence staining for MyHC in differentiated C2C12 cells transfected with pCDNA3.1-KLF4 vector. D. The fusion index (the percentage of nuclei in fused myotubes out of the total nuclei) in (C) was calculated. E. The mRNA levels of cell fusion related genes in KLF4 overexpression cells differentiated for 3 d. Data are represented as mean ± S.D. **P* < 0.05; ***P* < 0.01; ****P* < 0.001 (Student’s *t* test).
