# Supplementary figures and images for: KLF4 regulates skeletal muscle development and regeneration by directly targeting P57 and Myomixer
Source: Cell Death Dis. 2023 Sep 18;14(9):612. doi: 10.1038/s41419-023-06136-w (PMC10507053; doi:10.1038/s41419-023-06136-w)

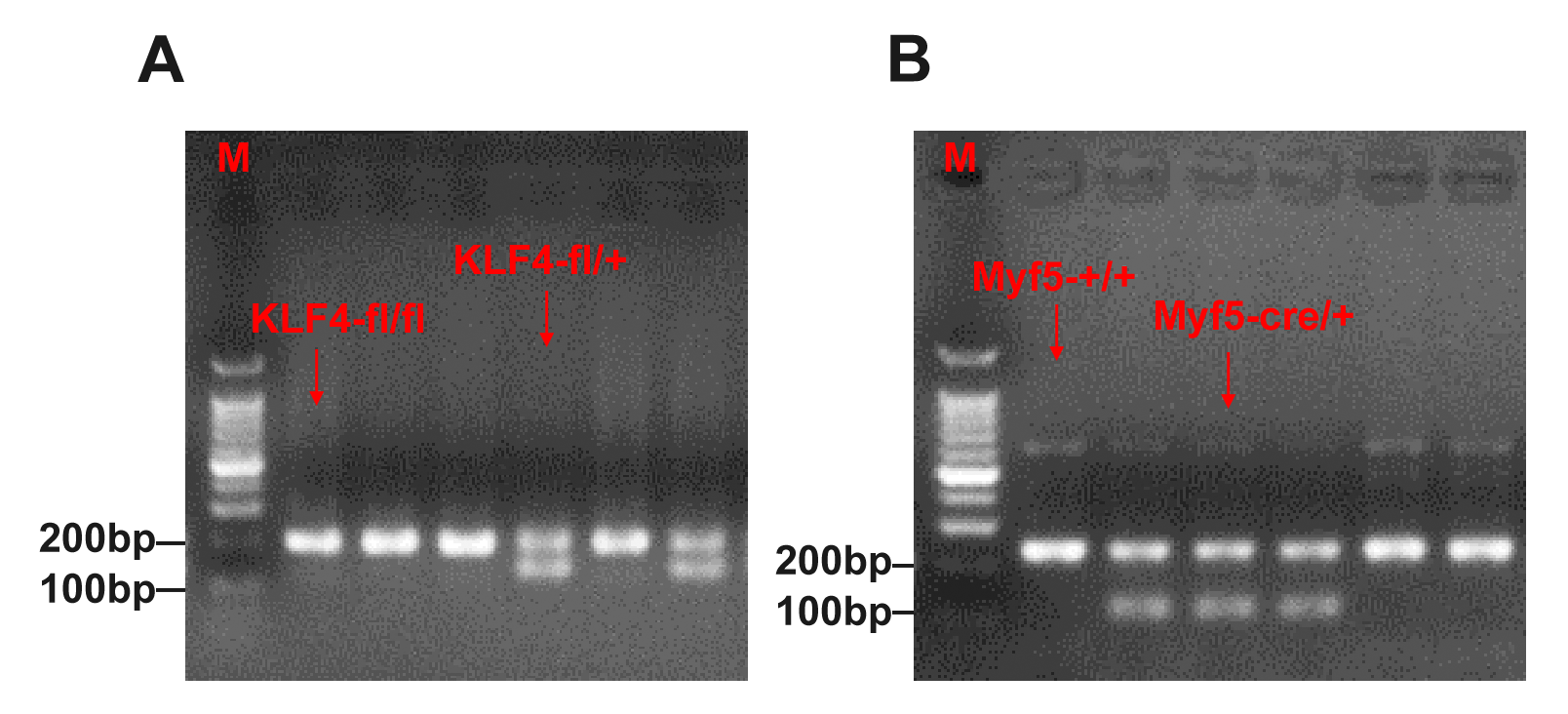

Supplement: Supplementary file 4 — Supplementary Figure 2 [file 41419_2023_6136_MOESM4_ESM.tif]

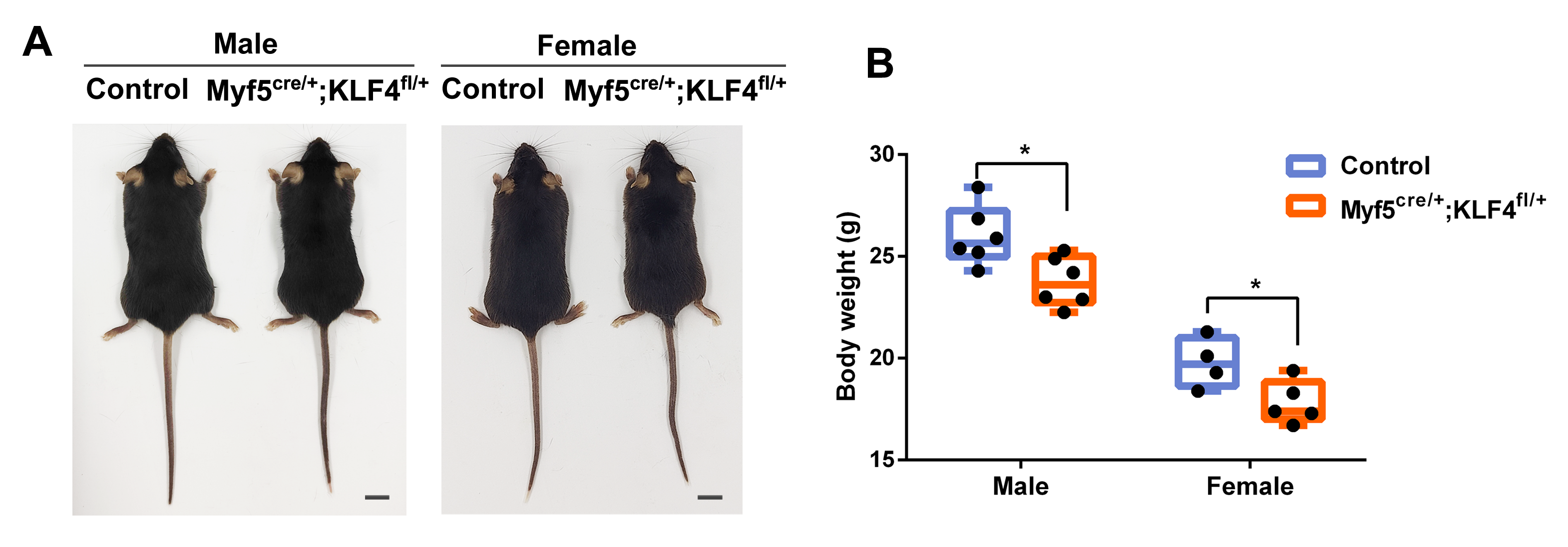

Supplement: Supplementary file 5 — Supplementary Figure 3 [file 41419_2023_6136_MOESM5_ESM.tif]

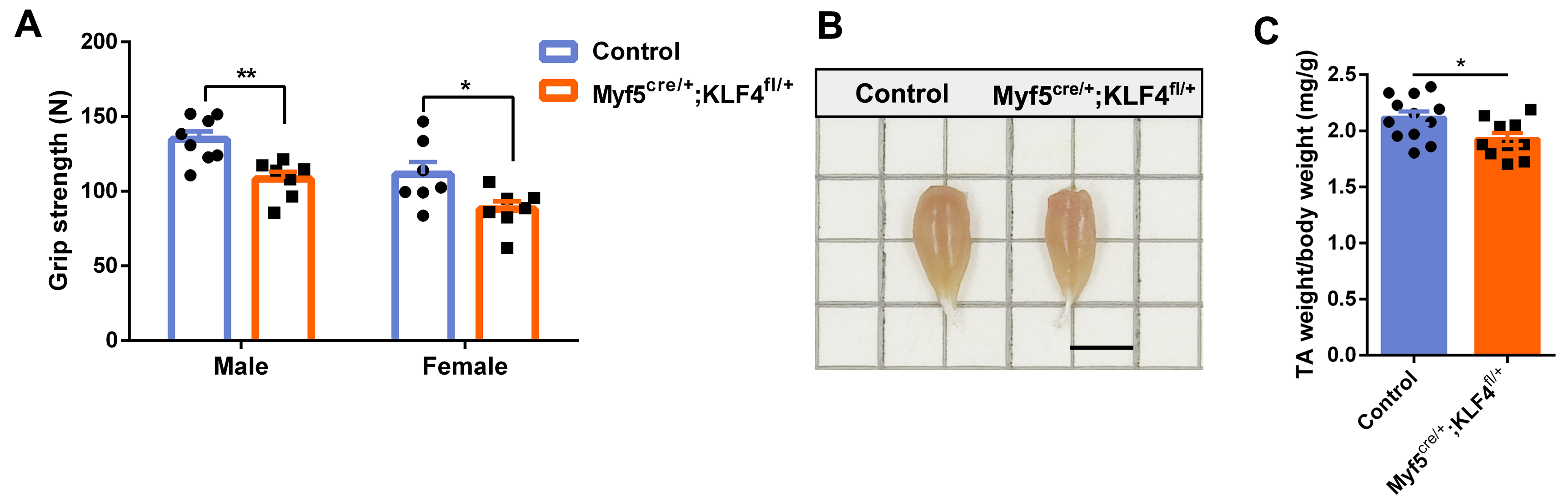

Supplement: Supplementary file 6 — Supplementary Figure 4 [file 41419_2023_6136_MOESM6_ESM.tif]

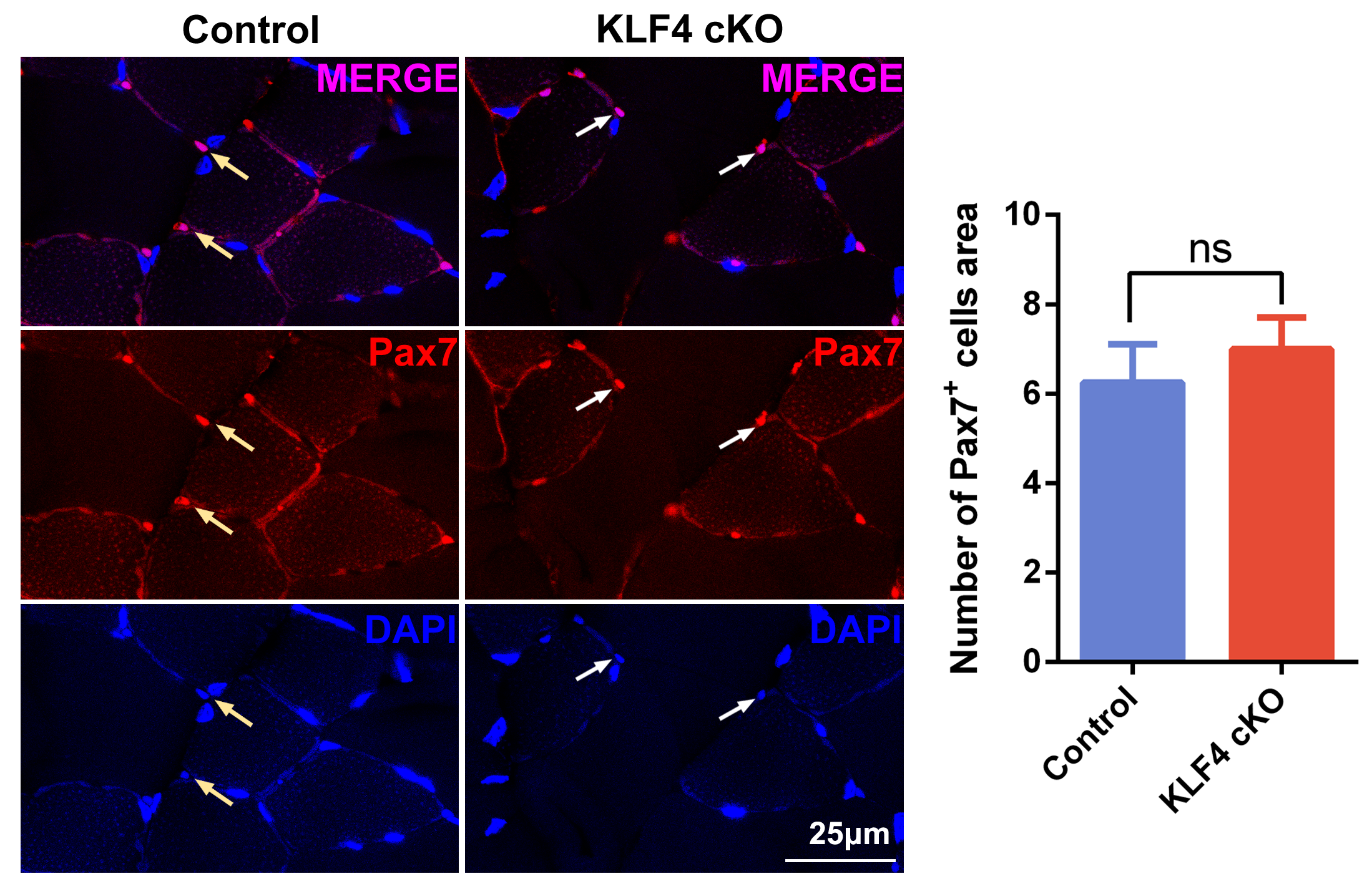

Supplement: Supplementary file 7 — Supplementary Figure 5 [file 41419_2023_6136_MOESM7_ESM.tif]

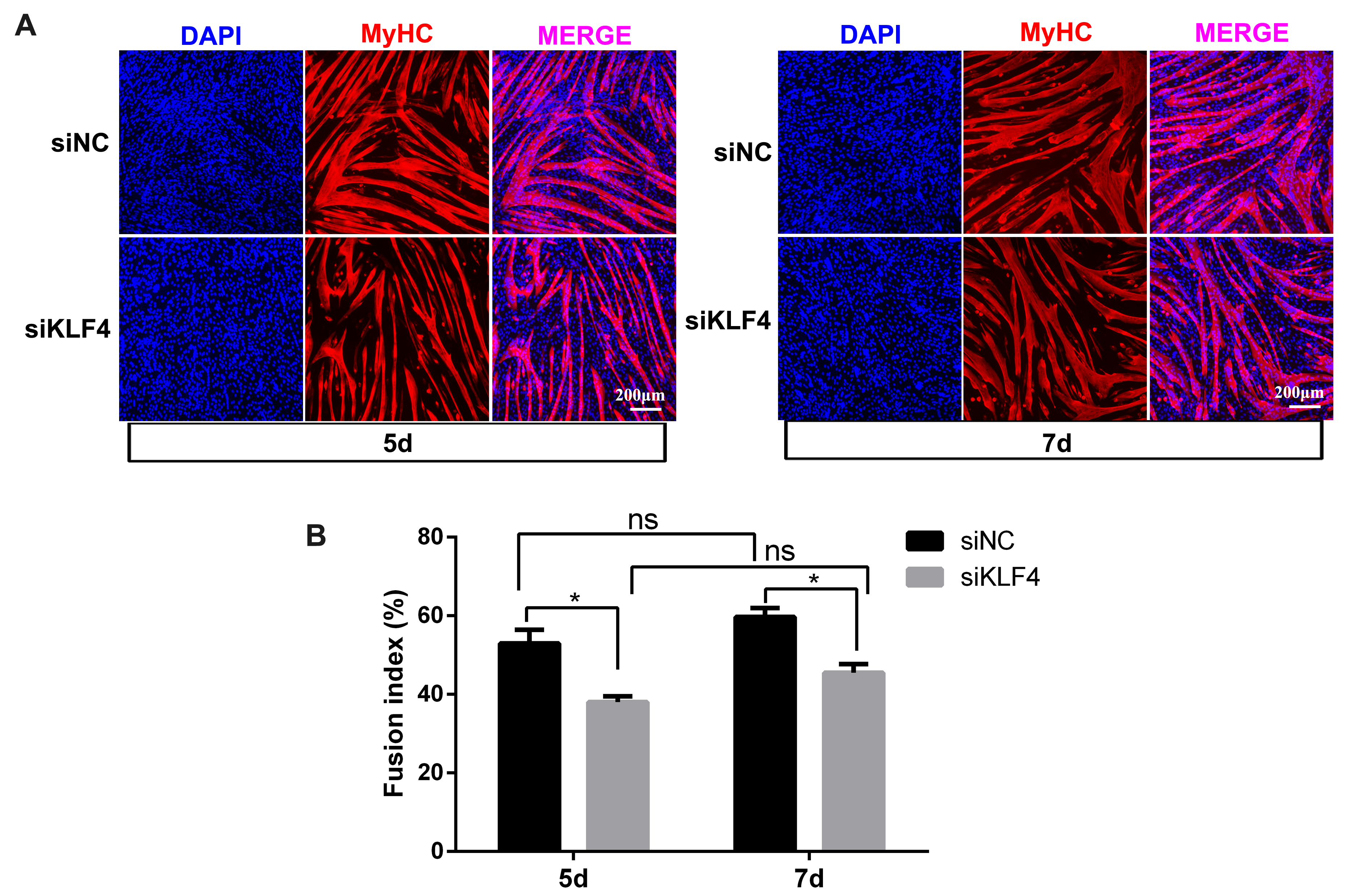

Supplement: Supplementary file 8 — Supplementary Figure 6 [file 41419_2023_6136_MOESM8_ESM.tif]

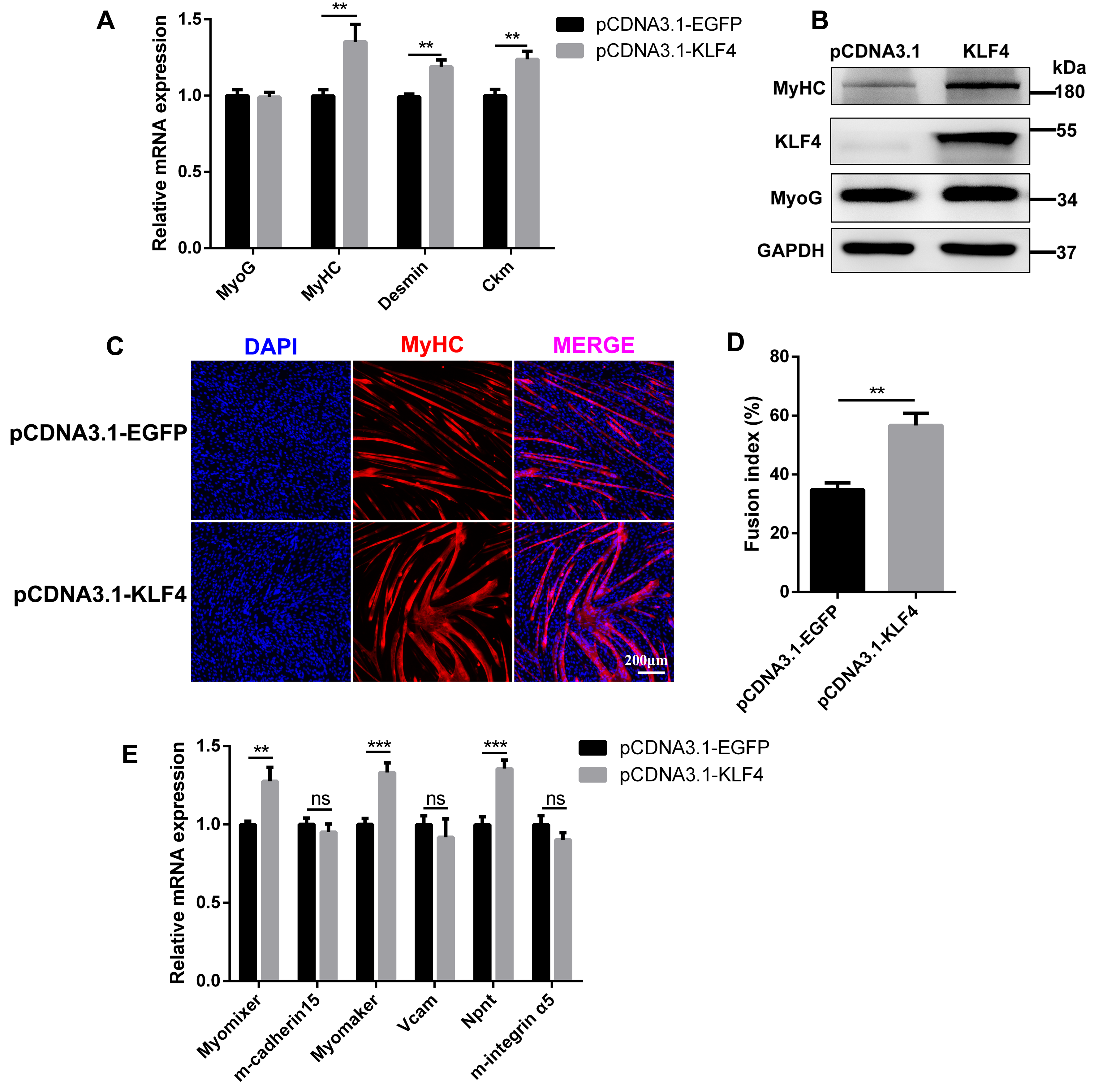

Supplement: Supplementary file 9 — Supplementary Figure 7 [file 41419_2023_6136_MOESM9_ESM.tif]
