# Supplementary material for: KLF4 regulates skeletal muscle development and regeneration by directly targeting P57 and Myomixer
Source: Cell Death Dis. 2023 Sep 18;14(9):612. doi: 10.1038/s41419-023-06136-w (PMC10507053; doi:10.1038/s41419-023-06136-w)
Supplement: Supplementary file 10 — Original Data File [file 41419_2023_6136_MOESM10_ESM.docx]

**Figure 1M: KLF4**

**
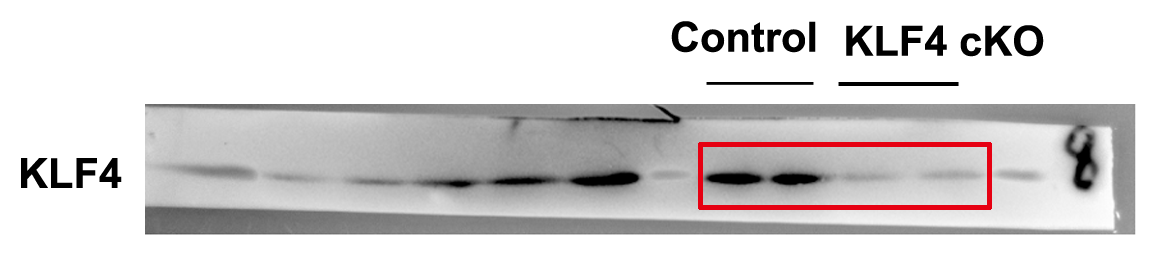
**

**Figure 1M: MyHC**

**
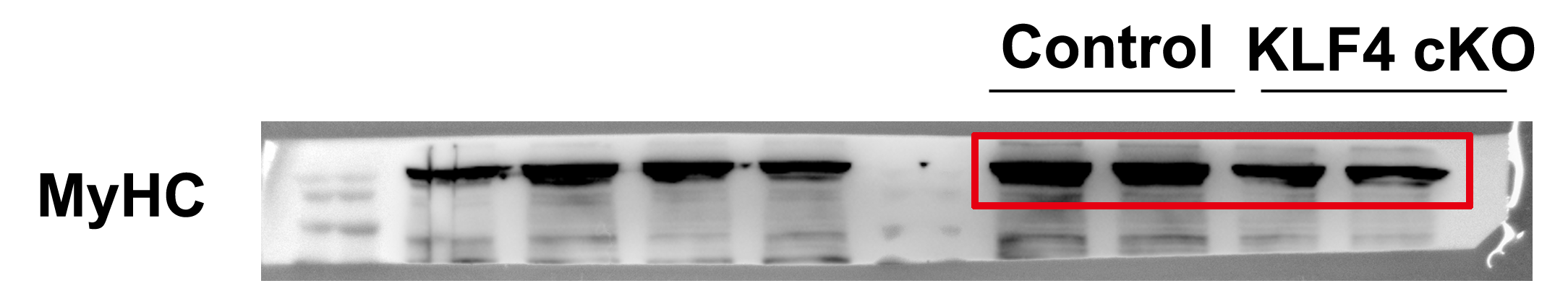
**

**Figure 1M: β-tubulin**

**
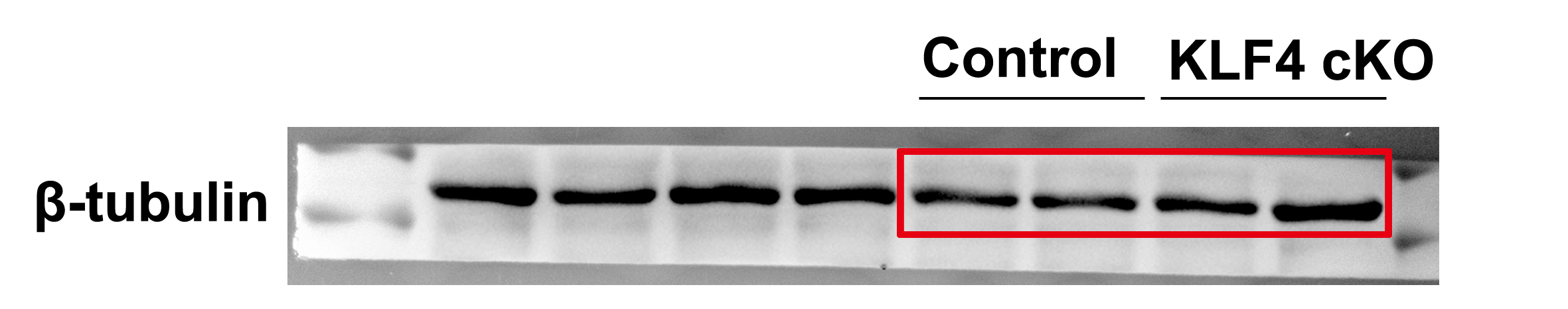
**

**Figure 2B**

**
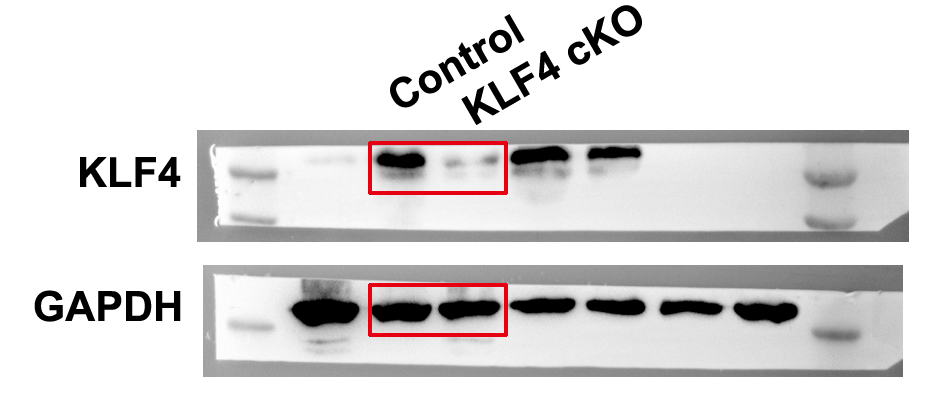
**

**Figure 3K: MyHC, P57, β-tubulin**

**
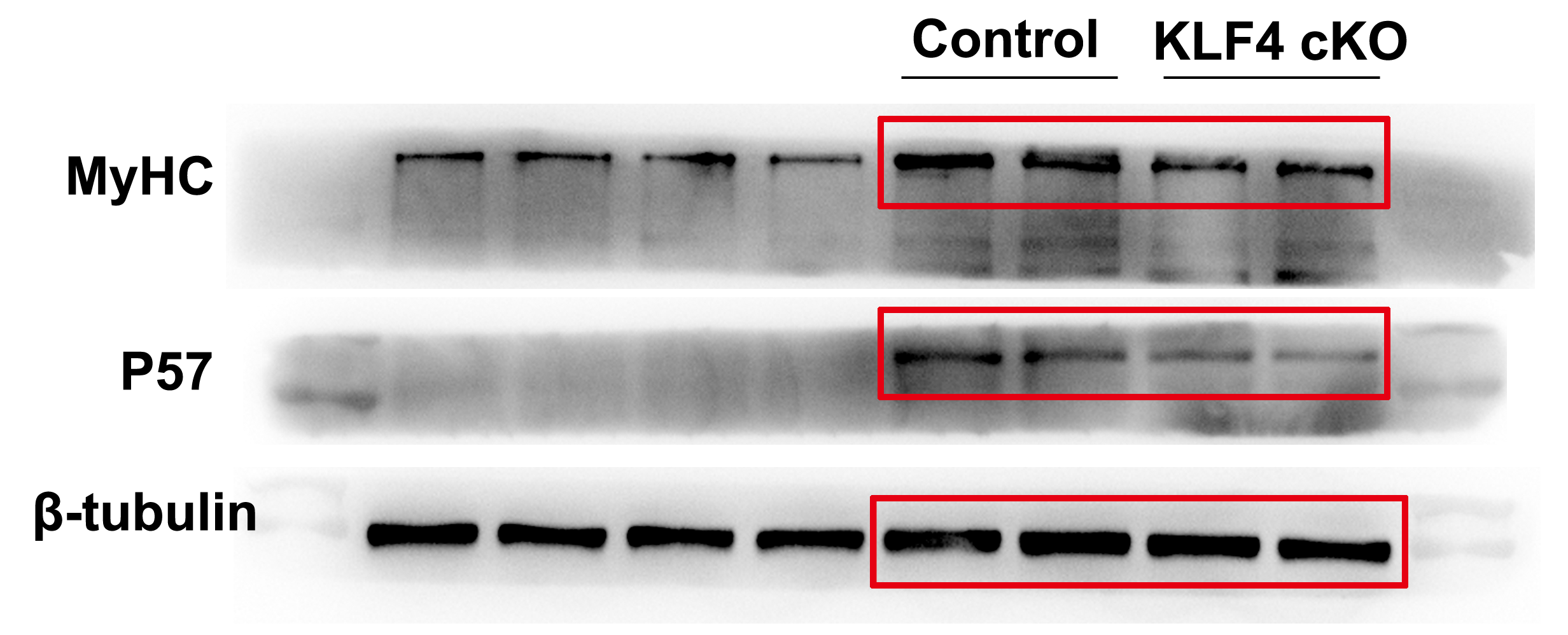
**

**Figure 3K: Myomixer**

**
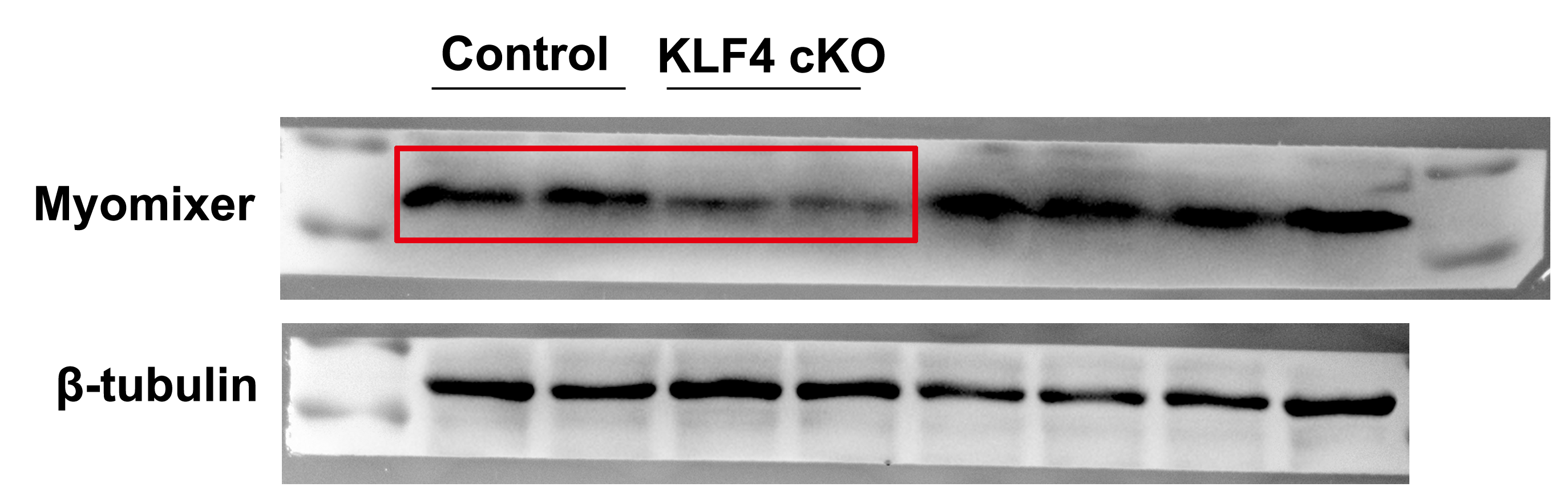
**

**Figure 3K: KLF4**

**
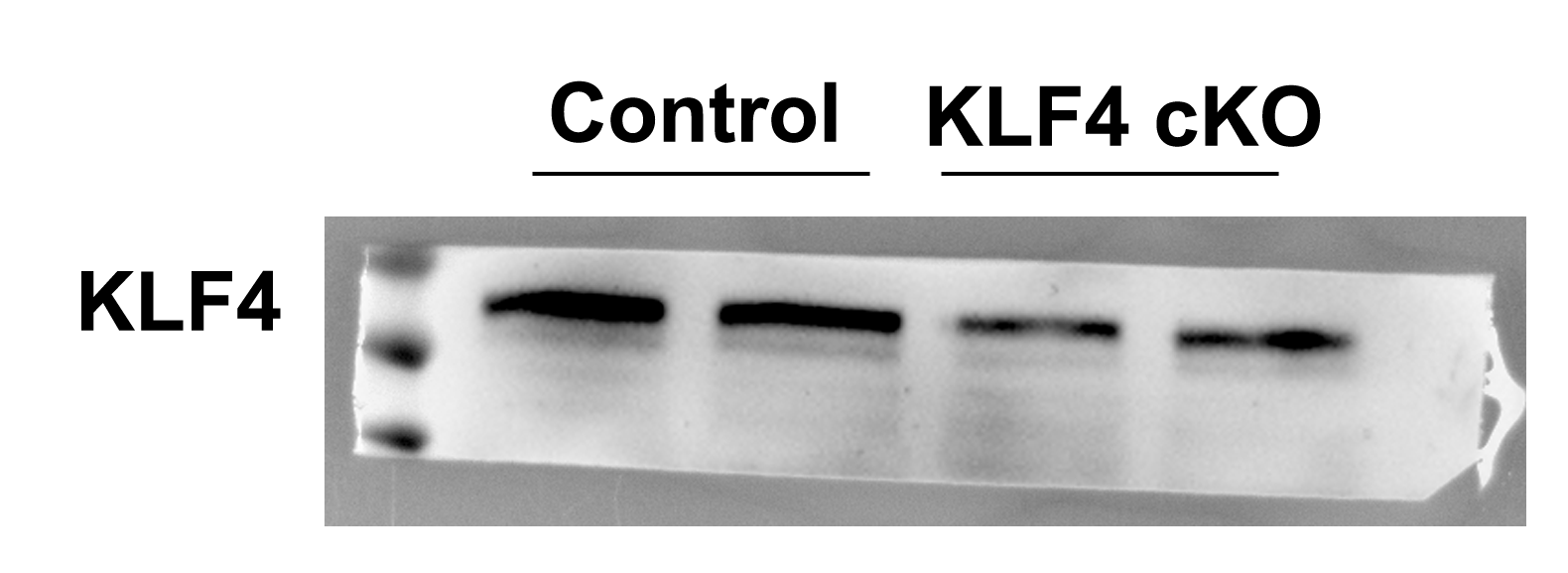
**

**Figure 3K: Cyclin D1**

**
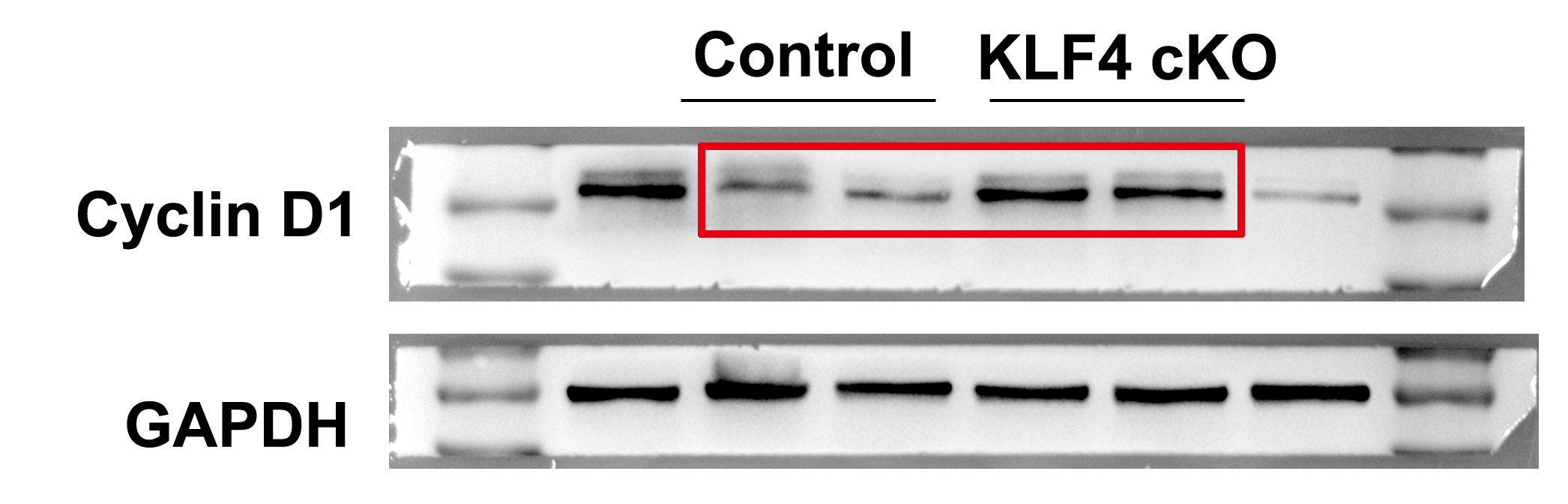
**

**Figure 4L: KLF4, GAPDH**


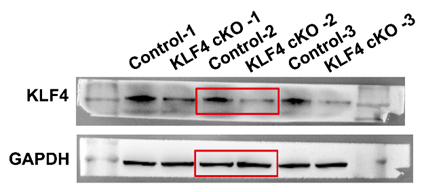


**Figure 4L: P57**


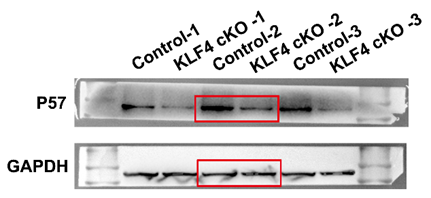


**Figure 4M: Myomixer, β-tubulin**


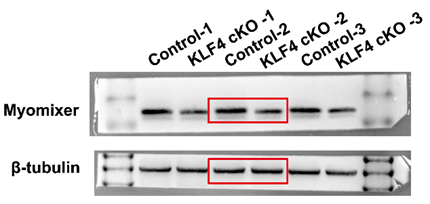


**Figure 5A**

**
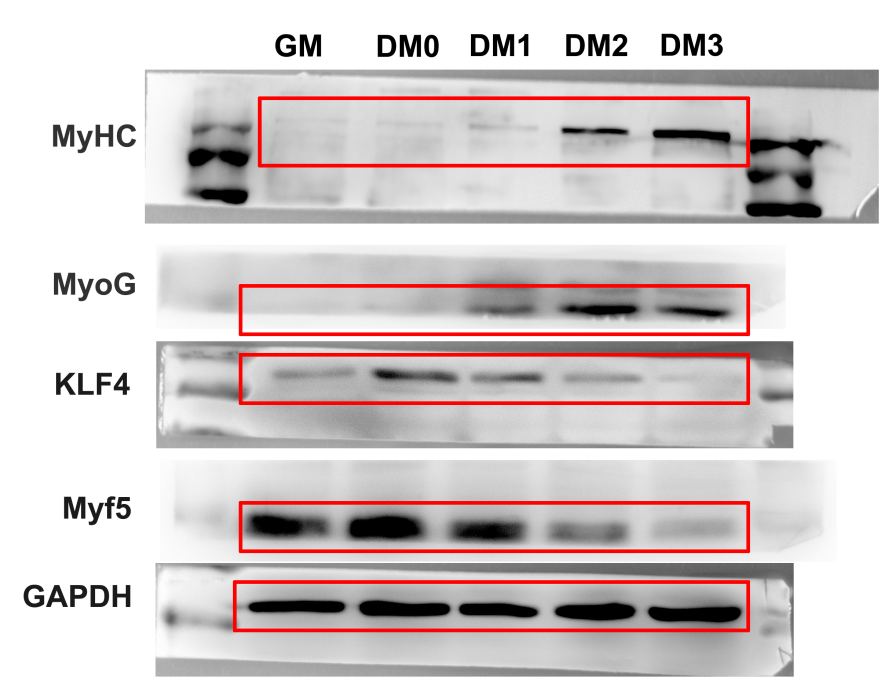
**

**Figure 5D**

**
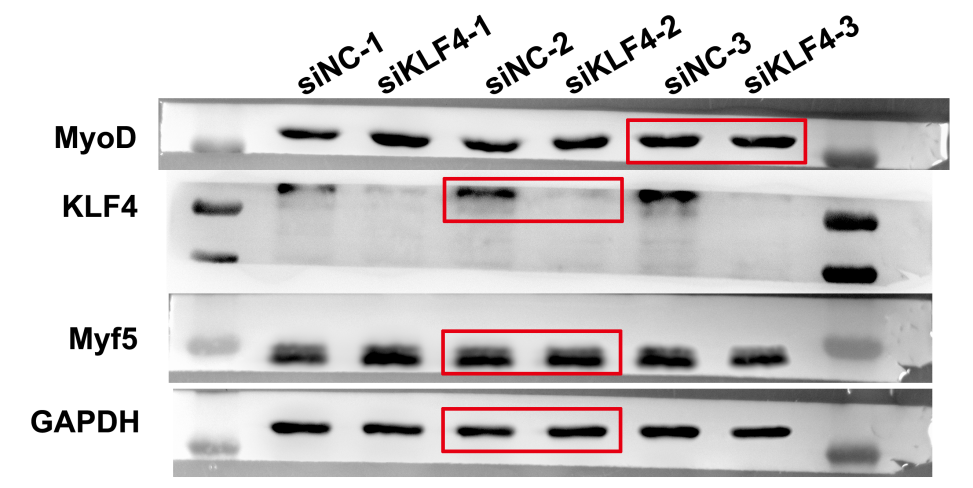
**

**Figure 5G: CyclinD1, P21, GAPDH**

**
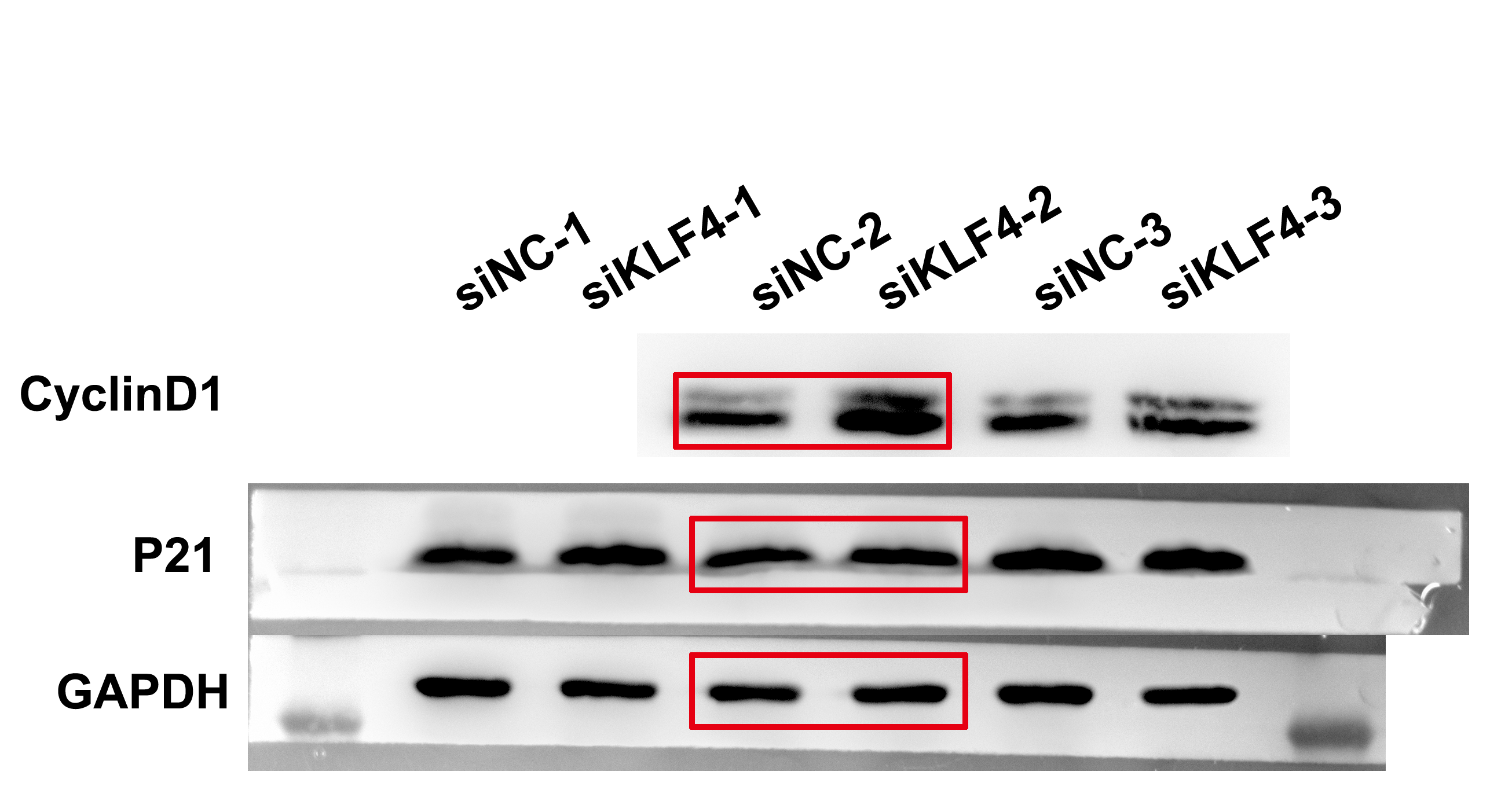
**

**Figure 5G: P57**

**
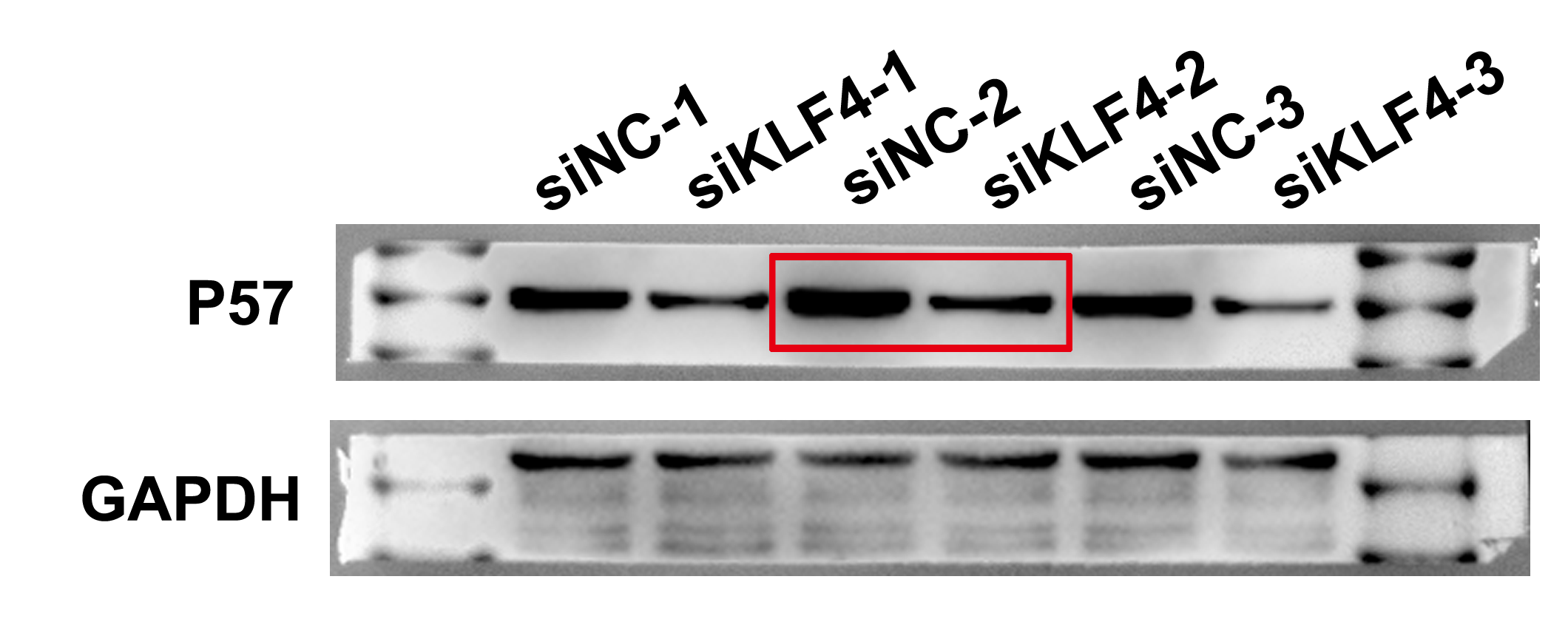
**

**Figure 6F: Cyclin D1**

**
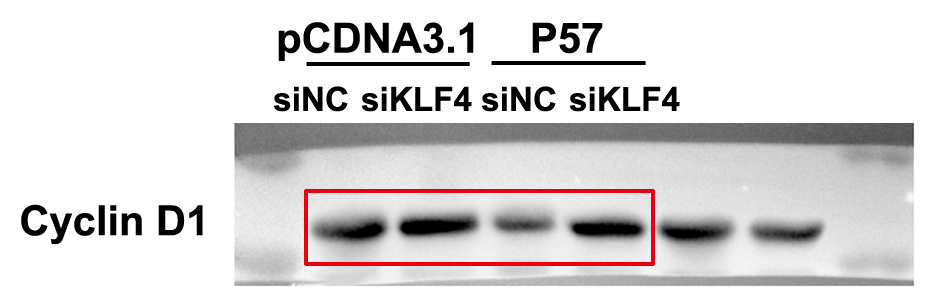
**

**Figure 6F: KLF4**

**
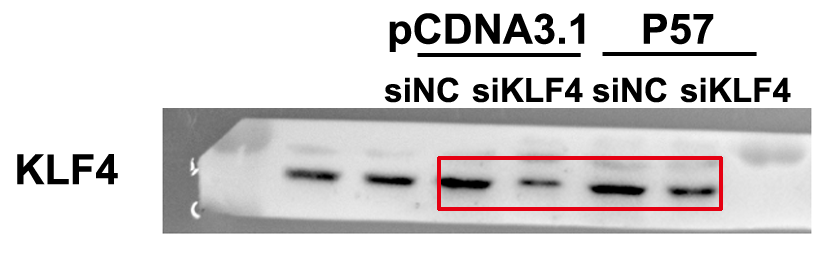
**

**Figure 6F: P57**

**
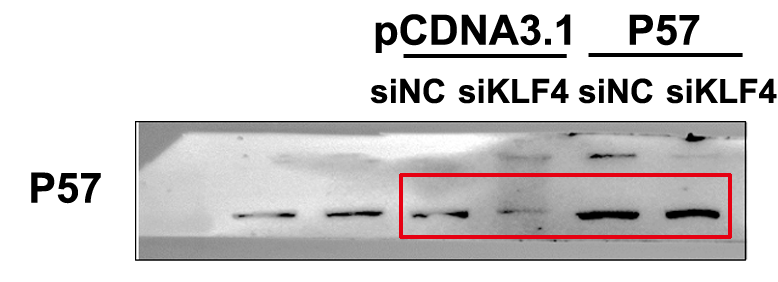
**

**Figure 6F: β-tubulin**

**
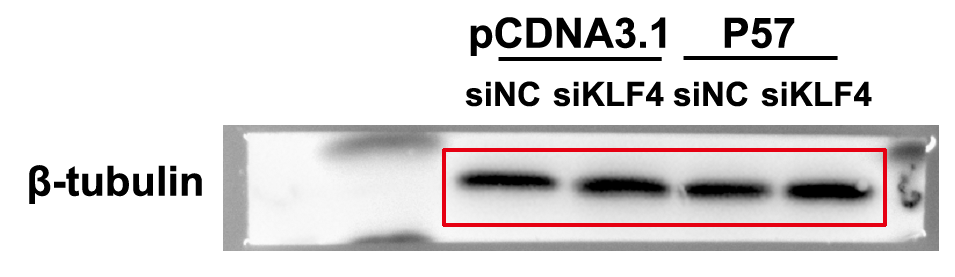
**

**Figure 7F**

**
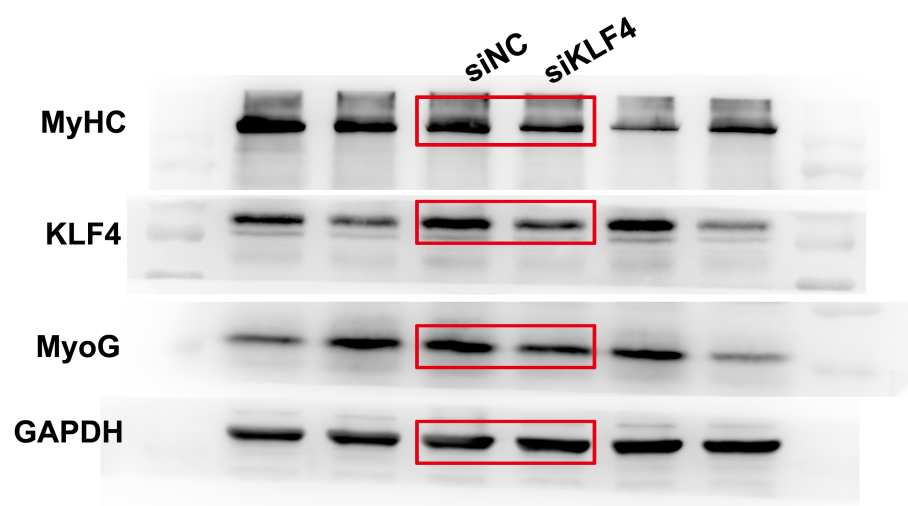
**

**Figure S6B**

**
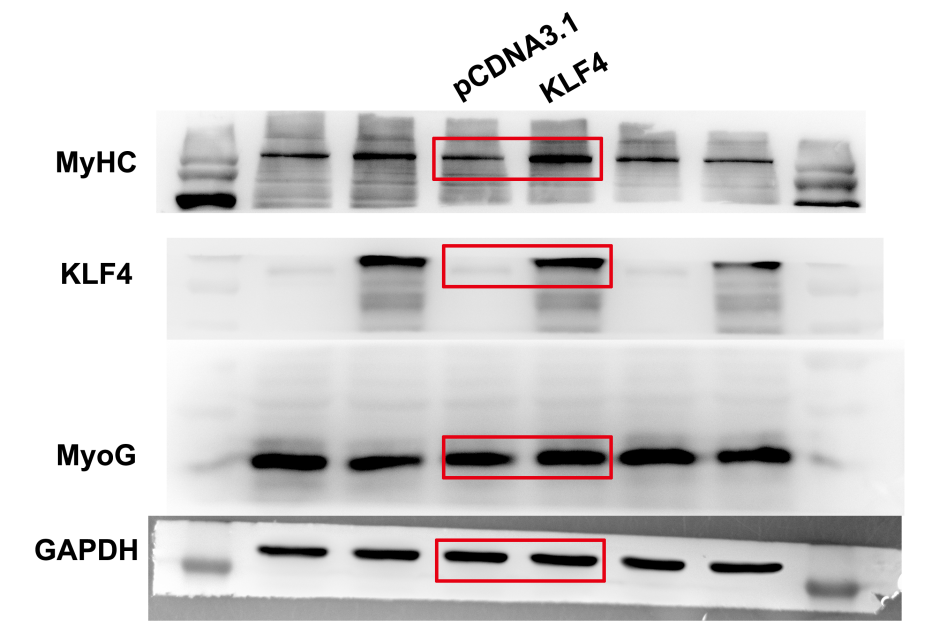
**

**Figure 8E**

**
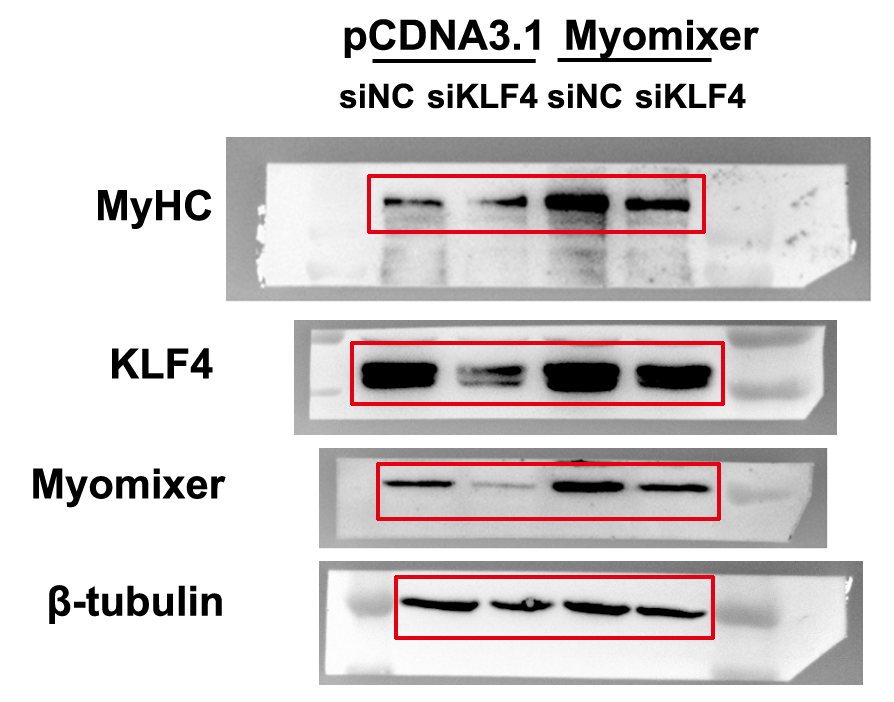
**
